# Epidemiology of neuropathic pain: an analysis of prevalence and associated factors in UK Biobank

**DOI:** 10.1097/PR9.0000000000001066

**Published:** 2023-03

**Authors:** Georgios Baskozos, Harry L Hébert, Mathilde MV Pascal, Andreas C. Themistocleous, Gary J Macfarlane, David Wynick, David LH Bennett, Blair H Smith

**Affiliations:** 1 Neural Injury Group, Nuffield Department of Clinical Neuroscience, John Radcliffe Hospital, University of Oxford, Oxford, UK; 2 Chronic Pain Research Group, Division of Population Health and Genomics, Mackenzie Building, Ninewells Hospital & Medical School, University of Dundee, Dundee, UK; 3 Epidemiology Group and Aberdeen Centre for Arthritis and Musculoskeletal Health, School of Medicine, Medical Sciences and Nutrition, University of Aberdeen, Aberdeen, UK; 4 Bristol Medical School, University of Bristol, Bristol, UK

## 1 Introduction

Chronic pain, usually defined as pain lasting for three months or more [53], is the leading cause of disability worldwide, when all pain conditions are taken into account, including low back pain, headache disorders and neck pain [23]. Neuropathic pain (NeuP) is a particularly severe form of chronic pain, arising as a direct consequence of a lesion or disease effecting the somatosensory nervous system [52]. Examples of common causes of NeuP include diabetes, HIV and chemotherapy treatment for cancer (causing painful peripheral neuropathies), herpes zoster (causing postherpetic neuralgia), multiple sclerosis, surgery, stroke and spinal cord injury [12]. NeuP is likely to be a major contributor to the global burden of chronic pain [7].

From a clinician’s perspective, it is important to distinguish NeuP from other forms of pain which arises from actual or threatened damage to non-neural peripheral tissue. NeuP is generally unresponsive to analgesics such as non-steroidal anti-inflammatory drugs or opioids. Rather gabapentinoids, tricyclic antidepressants and serotonin-norepinephrine reuptake inhibitors are recommended as first- and second-line treatments [18]. Nonetheless, these medications for NeuP provide greater than 50% pain relief in less than half of people treated. Furthermore, analgesics in general, particularly opioids and gabapentinoids, can potentially cause harm, providing an even greater emphasis on appropriate use [26].

In order to identify NeuP in the community screening tools such as the *Douleur Neuropathique en Quatre Questions* (DN4) [8], Self-Administered Leeds Assessment of Neuropathic Symptoms and Signs (S-LANSS) [5] and the painDETECT [20] have been developed. These rely on typical symptoms experienced in NeuP including burning, electric shocks, pins and needles and tingling. Estimates of NeuP in the general population suggest the prevalence is 7-10% [28], increasing to around 20-30% in people with diabetes [1,2,10,13]. Previous studies have also reported greater prevalence of NeuP, as with chronic pain overall, in older people, women and people from areas of high social deprivation [43].

However, due to differences in screening tools used and sample selection biases between studies, there is a large amount of heterogeneity in findings. Reliable information on NeuP prevalence and associated factors are vital for developing prevention and management strategies. The UK Biobank has recently re-phenotyped participants for chronic pain, creating, to the best of our knowledge, the largest available cohort for the study of pain, including NeuP. Although not representative of the general population, the size and scale of this cohort provides an ideal platform with which to validate the findings of previous studies and identify novel associations.

## 2 Methods

This study is reported using the Strengthening the Reporting of Observational Studies in Epidemiology (STROBE) guidelines for cross-sectional studies (Supplementary Table 1).

### 2.1 Dataset

#### 2.1.1 The UK Biobank cohort

UK Biobank comprises 501,518 volunteers recruited between 2006-10 (at ages 40-69 years) from across Great Britain. It consists of demographic and health data as well as linked bio-samples and specialist investigations such as imaging in subgroups [46]. A brief assessment of Chronic pain was completed by all participants at first assessment though it did not include validated questionnaires enabling categorisation of chronic pain and differentiation of NeuP from non-neuropathic pain (non-NeuP). The authors BS, DB, DW and GJM participated in the design of a set of revised UK Biobank pain questionnaires [6] between 2017 and 2018. This was based on validated questionnaires already in routine use and as far as possible aligned with established international consortia studying chronic pain such as Generation Scotland [41,42] and DOLORisk [27,40]. These were designed to focus on the most prevalent causes of chronic pain and associated co-morbidities and risk factors, including: musculoskeletal pain, NeuP and headache. The choice of items to include for NeuP was based on international recommendations made for phenotyping in genetic studies [29].

#### 2.1.2 Re-phenotyping for neuropathic pain

The chronic pain phenotyping survey was sent to all currently active UK Biobank participants in May 2019 who had consented to further electronic contact and had an active email address (n=335,587).

The pain questionnaires first asked about medical history, based on a list of conditions that commonly lead to chronic pain. These included musculoskeletal conditions such as osteoarthritis and rheumatoid arthritis but also important aetiological factors for NeuP such as diabetes, nerve damage, neuropathy and post-herpetic neuralgia.

Participants were asked to indicate if they had been suffering from pain for more than three months (defined as chronic pain [53]); if yes, they were asked if they experienced pain all over the body and if they answered yes they were asked to report pain intensity and directed to the American College Of Rheumatology (ACR) questionnaire screening for fibromyalgia [59]. If they did not experience pain all over the body they were directed to a list of body sites, where they indicated the locations in which they had experienced pain in the previous three months. They also rated the pain intensity at each location over the last 24 hours, on an 11-point visual analogue scale (VAS). They then identified which of these pains had bothered them most during the previous three months. The subsequent pain-related questionnaires asked the participants to complete them with regard to their most bothersome pain. Participants completed the self-reported items on the DN4 questionnaire which is a validated tool for NeuP screening based on pain quality [8]. The characteristics of the most bothersome pain were captured with bespoke questions based on the Brief Pain Inventory (BPI) – Short Form [11], asking about pain intensity over the previous 24 hours and on average. Participants were asked to rate the relief they obtained from pain medication, and how their pain impacted different areas of their lives.

The EQ-5D 5 levels (EQ-5D-5L)[30] was used to ask about health-related quality of life in more detail; this included a 0-100 VAS that represented the participant’s health at that time, and an assessment of the extent of limitations experienced in different domains of life (mobility, self-care, usual activities, pain or discomfort, and anxiety and depression).

The presence of peripheral neuropathy in the legs and feet was assessed with the self-complete component of the Michigan Neuropathy Screening Instrument (MNSI) [16], for those participants who answered yes to the presence of at least one of the following in the medical history section: cancer pain, diabetes or nerve damage other than diabetic neuropathy. These conditions are common causes of length-dependent peripheral neuropathy, and symptoms are expected to be present distally. Finally, participants were asked to complete psychosocial questionnaires about depression (Patient Health Questionnaire-9, PHQ-9 [35]) and fatigue (Fatigue Severity Scale, FSS [36]).

Body Mass Index (BMI) was calculated from data measured during the initial Assessment Centre visit in 2006-2010. Ethnic background, job and current employment status were also collected at recruitment. Age of participants at the time of completion of the pain phenotyping questionnaires was calculated from the year of birth, collected at recruitment, and the time the questionnaires were completed. Index of Multiple Deprivation was calculated using data from distinct domain of deprivations collected at recruitment. The distinct primary diagnosis, coded using the International Classification of Diseases (ICD-10), were recorded from the participants hospital inpatient record during 2013-2022. Full background information about the follow-up UK Biobank pain questionnaire, and the questions included is available at https://biobank.ctsu.ox.ac.uk/crystal/ukb/docs/pain_questionnaire.pdf.

For analysing this study, we divided the cohort into three groups:

1) Chronic neuropathic pain (NeuP): participants who reported having pain for more than three months AND who scored 3 or more on the DN4, N = 13,744
2) Chronic non-neuropathic pain (Non-NeuP): participants who reported having pain for more than three months AND who scored less than 3 on the DN4, N = 62,351
3) No chronic pain (NoCP): participants who did not report having pain, OR whose reported pain had lasted less than three months, N = 72,733

Only fully completed DN4 questionnaires were considered for group allocation. Chronic pain participants with an incomplete DN4 were not classified. A flowchart of the group definition is in supplementary figure 1.

### 2.2 Statistical analysis

Data was collected electronically by completion of a structured online questionnaire. As non-white ethnic backgrounds were rare in UK Biobank (2.2%), we grouped together all Black, Asian and Minority ethnicities (BAME) into one group, noting the lack of homogeneity of this group. Of the total UK Biobank cohort, 1.7% were BAME, 0.5% were mixed ethnicity and the remaining 97.3% were white.

Occupations were encoded according to the Departments of National Statistics Standard Occupational Classification (SOC 2000) major groups. The Index of Multiple Deprivation, a weighted composite score showing relative deprivation, i.e. the higher the score the more deprived the individual, was used as a measure of poverty.

In order to determine differences between groups we carried out one-way ANOVA for normally distributed data or the Kruskal Wallis test for non-parametric data. These were followed up with Tukey’s HSD post-hoc tests. Associations between groups and categorical variables were tested using the Chi-Square test and followed up by pairwise Bonferroni corrected chi-square post-hoc tests. Statistical significance cut-off was p.value < 0.05.

In addition, we carried out multivariable modelling using binomial or multinomial, multiple logit regression depending on the number of levels of the dependent variable. Before model fitting, we removed variables with >30% missing values, low variance (ratio cut-off 98/2, unique < 10), the least informative variable (i.e. lower variance) from highly correlated pairs (Pearson’s correlation coefficient >0.8) or those directly measuring pain, missing values were imputed in 20 cycles of multiple imputations by chained equations using the predictive mean matching algorithm. Missing value percentages for all variables are in supplementary table 2. We considered a set of uncorrelated independent variables that included the EQ5D index, sex, age, job, index of multiple deprivations, the 3 first principal components of genetic variation and binary tags for self-reported diseases: Diabetes, Other Neuropathy, Osteoarthritis, Rheumatoid arthritis, Cancer pain, Carpal Tunnel Syndrome, Chronic post-surgical pain, Gout, Migraine, Pelvic pain. The genetic variation principal component loadings were downloaded from UK Biobank and were calculated by PCA analysis on 101284 SNPs.

Model coefficients were aggregated across imputations using Rubin’s rules. For the significant model coefficients (one-way ANOVA p.value <0.05), we present odds ratios and the associated 95% confidence intervals.

## 3 Results

### 3.1 Demographics and socio-economic status

**Figure 1:**
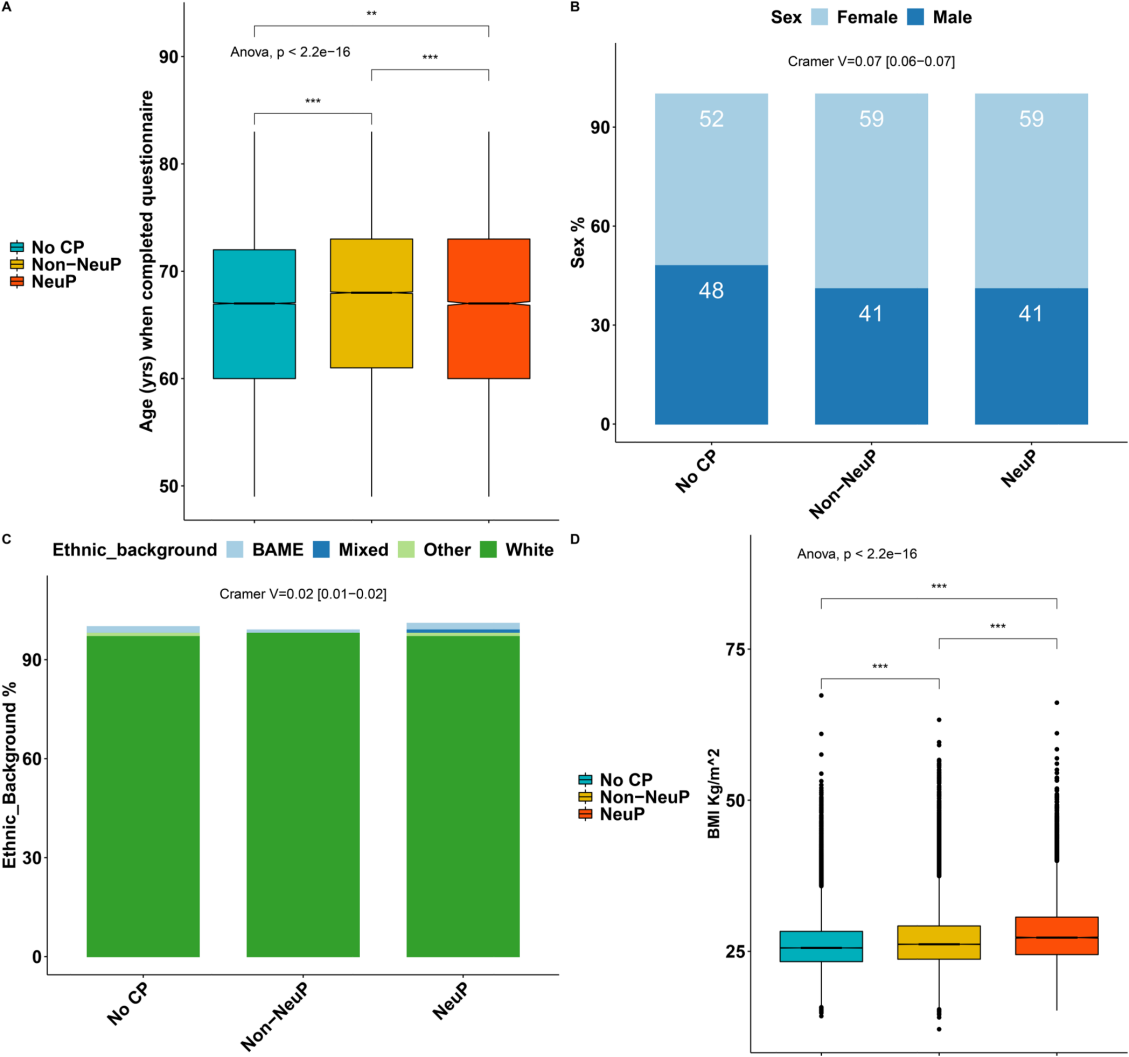
Demographics of participants with chronic NeuP, Non-Neup and NoCP. A: Boxplots show the age of participants when questionnaires were completed for each group. Notched line represents the median. B: Stacked bar plots show the sex distribution across the three groups. C: Stacked bar plots show the ethnic background distribution across the three groups. D: Boxplots show the BMI of participants across the three groups. Omnibus ANOVAs are labelled in the plot and are followed-up by t-tests between groups. P.value < 0.001 is coded as ***, p.value < 0.01 is coded as ** 0.01, p.value < 0.05 is coded as *. Post-hoc tests are shown in supplementary figure 2.

**Figure 2:**
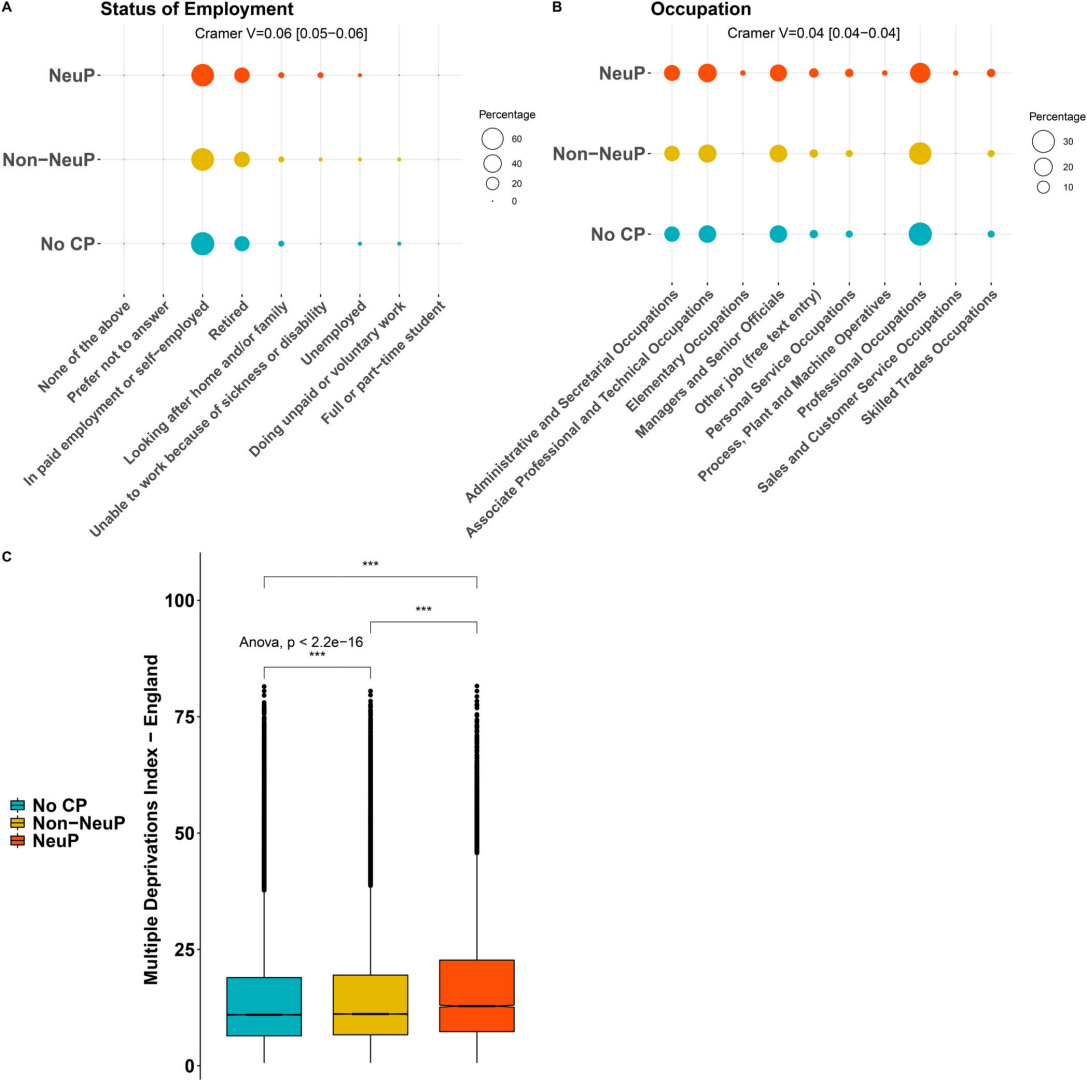
Socio-economic status of participants with chronic NeuP, Non-Neup and NoCP. A: Dotplot shows the employment status of participants for each group. The group is colour coded, the respective percentage is coded in the size of the dot. B: Dotplot shows the occupation of participants for each group. C: Boxplots show the index of multiple deprivations across the thee groups. Omnibus ANOVAs are labelled In the plot and are followed-up by t-tests between groups. P.value < 0.001 is coded as ***, p.value < 0.01 is coded as ** 0.01, p.value < 0.05 is coded as *. Post-hoc tests are shown in supplementary figure 3.

167,203 participants completed the pain experience phenotyping (response rate 49.8%). There was an over-representation of participants who were female, of younger age, lower BMI and less socially deprived in the group that chose to complete the pain re-phenotyping questionnaire compared to the rest of the UK Biobank cohort, regardless of whether they were sent a questionnaire, supplementary table 3. 148,828 out of the 167,203 (89%) participants had no chronic pain or chronic pain with a fully completed DN4 questionnaire (see methods). The participants with completed DN4 had the same deprivation levels as those that had not completed the questionnaire, but there were more females, of slightly higher age and higher BMI than the participants with incomplete DN4, supplementary table 4. 13,744 participants reported chronic NeuP; 62,351 participants reported chronic Non-NeuP; 72,733 participants reported no chronic pain. The prevalence of NeuP in this cross-sectional cohort was therefore 9.2% (13734/148828). Age and sex showed weak significant associations with outcome: participants with NeuP were younger than those with Non-NeuP (Means Difference (MD) –0.33, 95%CI[-0.5, -0.16]), figure 1A and supplementary figure 2A. Participants who reported chronic pain, both Neuropathic (NeuP) and Non-Neuropathic (Non-NeuP), were more likely to be female than those participants without chronic pain (59% vs 52%), figure 1B and supplementary figure 2B. Ethnic background was significantly but weakly associated with outcome, figure 1C. 96.7% of participants with NeuP were white British; 97.3% in the total UK Biobank cohort. There was a higher proportion of whites in the Non-NeuP (97.7%) vs the other groups, while BAME were more likely to report NoCP (1.8%) and NeuP (1.9%) versus Non-NeuP (1.4%), supplementary figure 2C. BMI was significantly associated with pain grouping (p.value <0.001): BMI was significantly higher in participants with NeuP vs Non-NeuP, MD 1.14, 95%CI[1.04, 1.23]) and in participants with Non-NeuP vs NoCP (MD 1.86, 95%CI[1.76, 1.96]), supplementary figure 2D. Both employment status and occupation/job type were significantly but weakly associated with the outcome. People with NeuP were more likely to report that they were “Unable to work because of sickness or disability” compared to those with Non-NeuP and NoCP, figure 2A and supplementary figure 3A. People with NeuP were more likely to work in “Elementary occupations”- simple and routine tasks, involving the use of hand-held tools and in some cases considerable physical effort; “Process, plant and machine operatives” - operate and monitor large scale, industrial machinery and equipment; and “Personal service occupations” compared with the other two groups, figure 2B and supplementary figure 3B. The multiple deprivation index was significantly associated with the outcome and was higher (consistent with increased deprivation), in people with NeuP vs Non-NeuP (MD 2.04, 95%CI[1.76, 2.32]) and in NeuP vs NoCP (MD 2.34, 95%CI[2.06, 2.61]). It was also slightly higher in and Non-NeuP vs NoCP (MD 0.3, 95%CI[0.14, 0.46]), supplementary figure 3C.

### 3.2 Pain rating and quality of life

**Figure 3:**
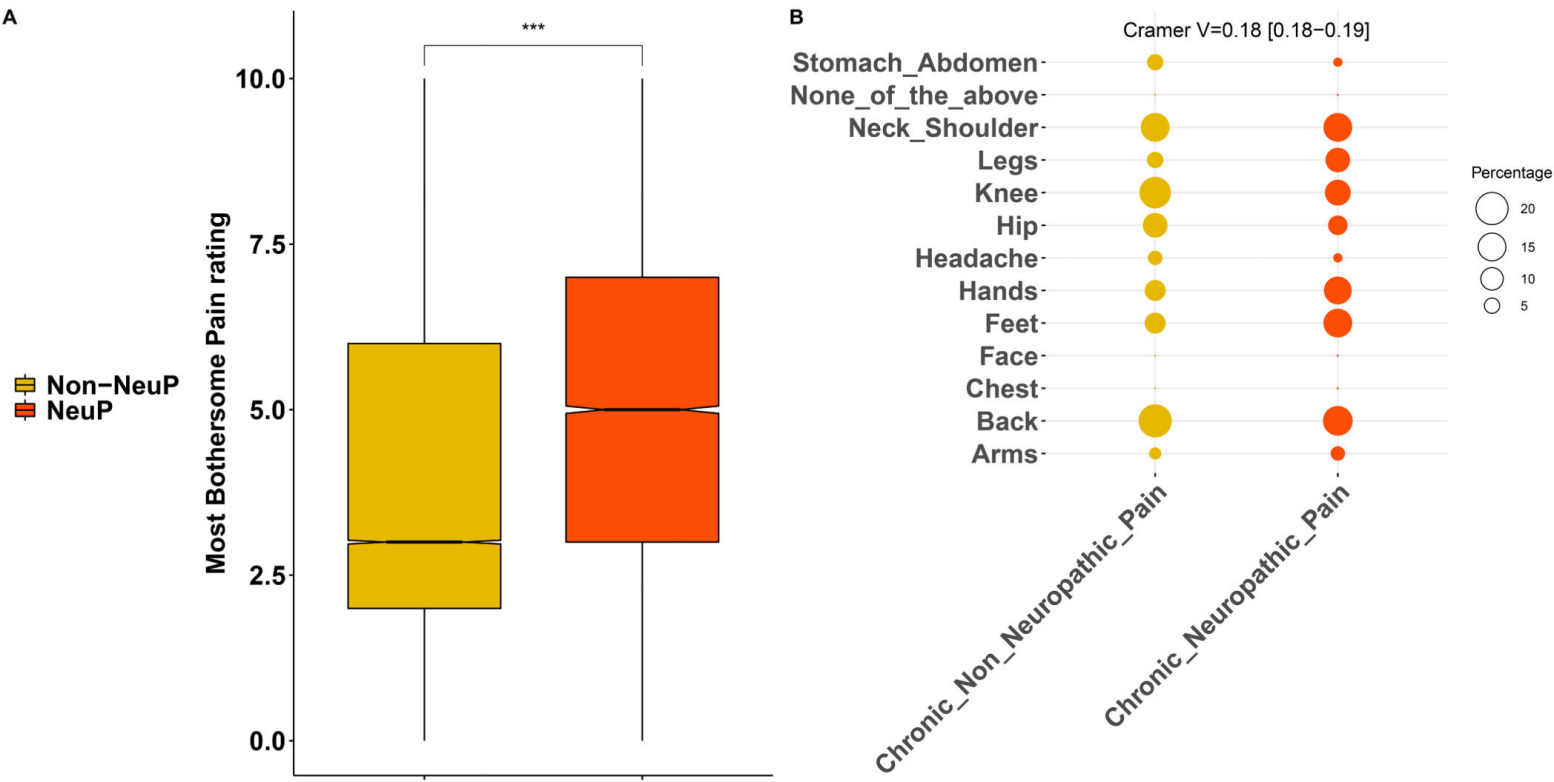
Pain rating and location of the most bothersome pain. A: Boxplots show the pain rating for the self-reported location of the most bothersome pain for both painful groups, NeuP vs Non-NeuP. B: Dotplot shows the frequencies for the self-reported location of the most bothersome pain for the two painful groups. Rates are coded in the size of the dot. P.value < 0.001 is coded as ***, p.value < 0.01 is coded as ** 0.01, p.value < 0.05 is coded as *.

**Figure 4:**
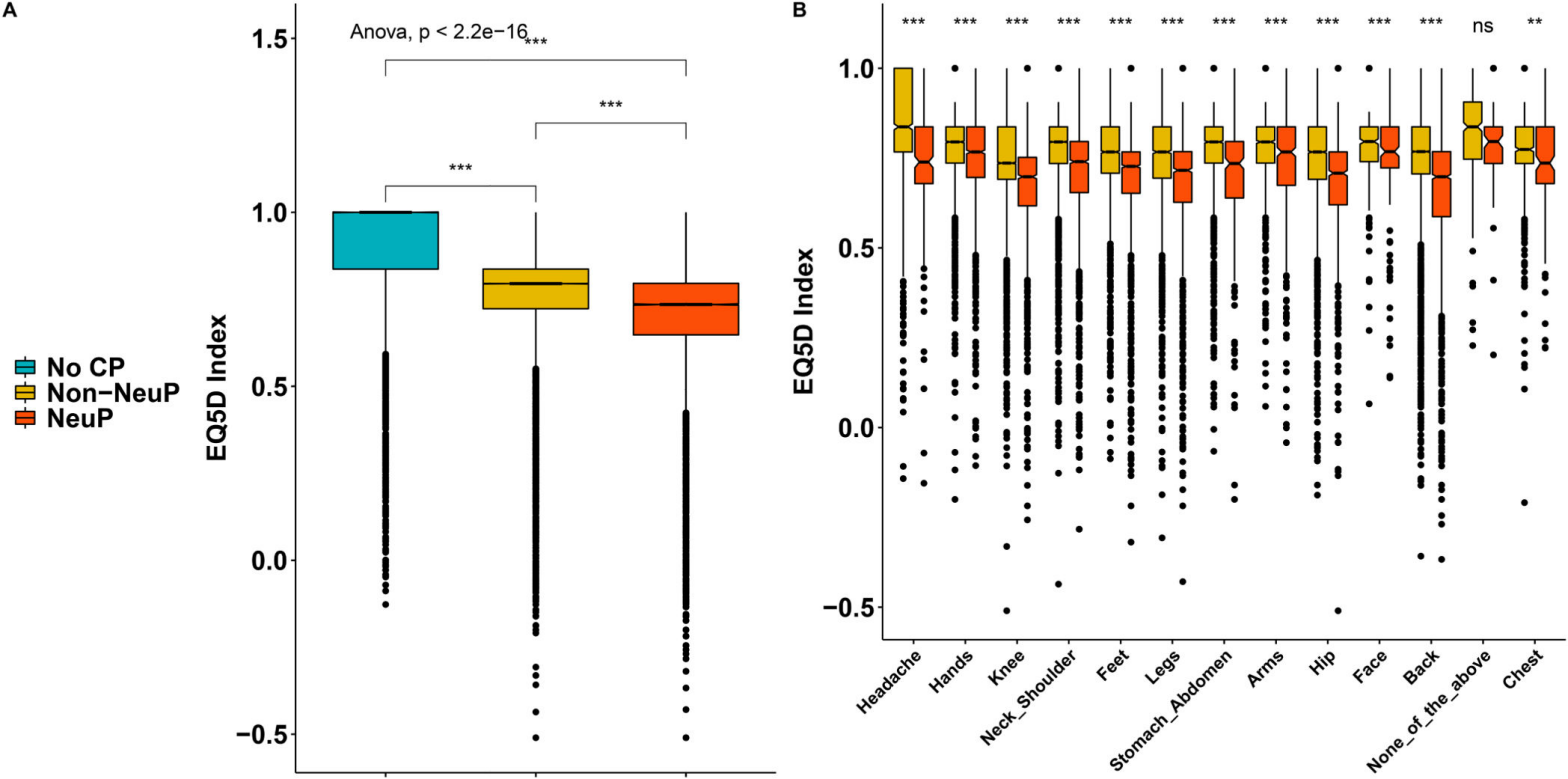
The impact of pain in quality of life. A: Boxplots show the EQ5D index across the three groups. B: Boxplots show the EQ5D for both painful groups across all locations reported as the ones having the most bothersome pain. Omnibus ANOVAs are labelled In the plot and are followed-up by t-tests between groups. P.value < 0.001 is coded as ***, p.value < 0.01 is coded as ** 0.01, p.value < 0.05 is coded as *. Post-hoc tests are shown in supplementary figure 4.

**Figure 5:**
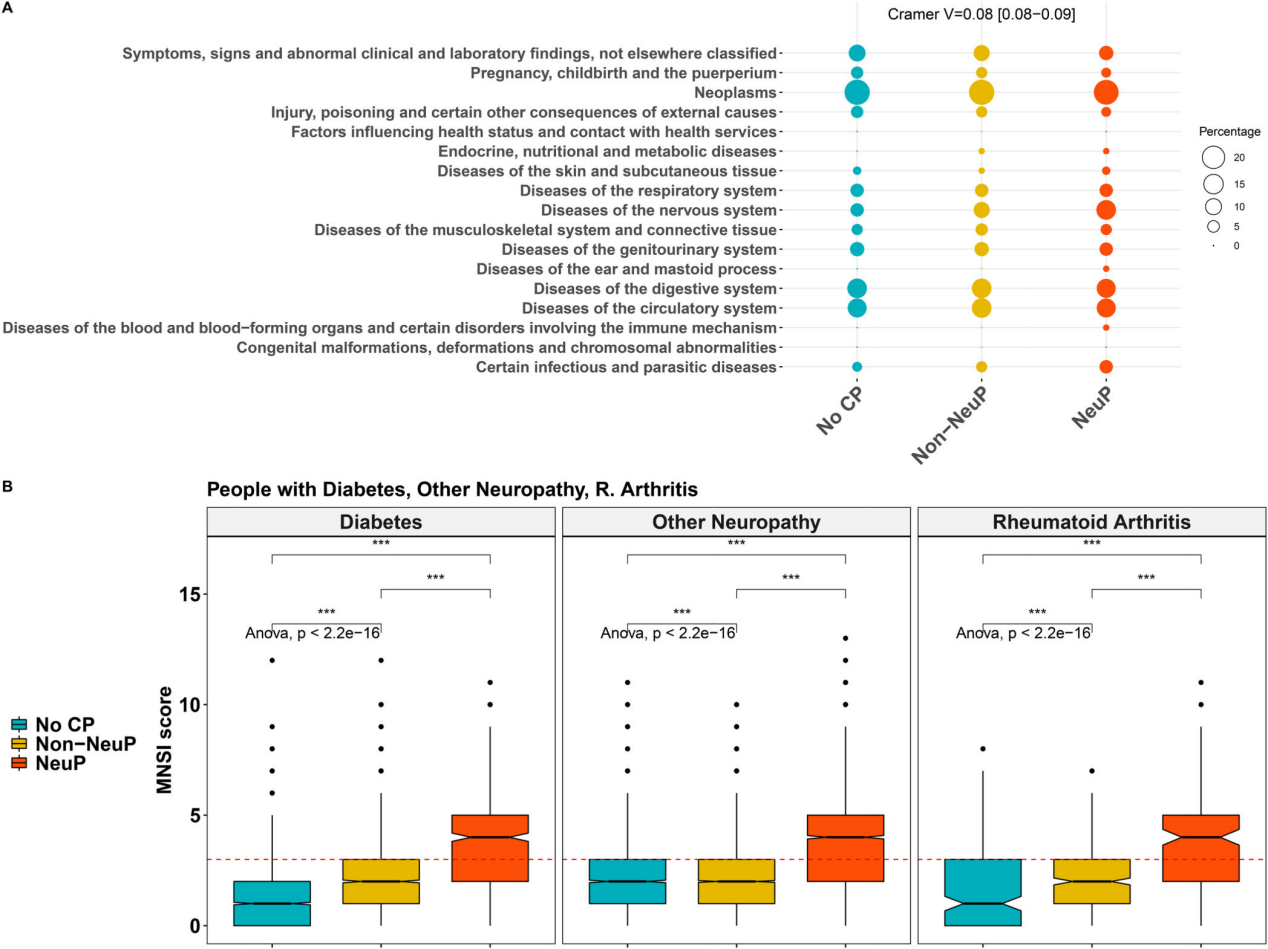
A:Frequencies of diseases across the three groups. The rates are coded in the size of the dot. Group allocation is colour coded. Post-hoc tests are shown in supplementary figure 5. B: MNSI scores across the three groups in participans with diabetes, rheumatoid arthritis and other neuropathy.

People with NeuP reported higher pain severity ratings for the self-reported most bothersome pain, figure 3A. The most bothersome pain in people with NeuP was reported to be in the feet, hands, or legs more frequently, whereas the most bothersome pain was reported to be in the back, hip, knee, stomach, abdomen, or head most frequently by people with non-NeuP, figure 3B. The quality of life as measured by the EQ5D normalised index was dependent on outcome and was significantly lower in people with NeuP vs NoCP (MD –0.22, 95%CI[-0.23, -0.22]) and Non-NeuP vs NoCP (MD –0.14, 95%CI[-0.14, -0.14]), figure 4A and supplementary figure 4. The association between NeuP and reduced quality of life was consistent across all body locations figure 4B. People with NeuP were more likely to have “Diseases of the nervous system” than by both the other groups and “Certain infectious and parasitic diseases” than people with NoCP, figure 5 and supplementary figure 5. People with Diabetes, Rheumatoid Arthritis (RA) or Other Neuropathy, were considered at risk of peripheral neuropathy and have completed the Michigan Neuropathy Screening Instrument (MNSI), figure 5B. People with NeuP had higher MNSI scores than both NoCP and Non-NeuP. Only people with NeuP had a score of 3 and above, which is a reasonable cut-off threshold for peripheral neuropathy, in their interquantile range.

**Figure 6:**
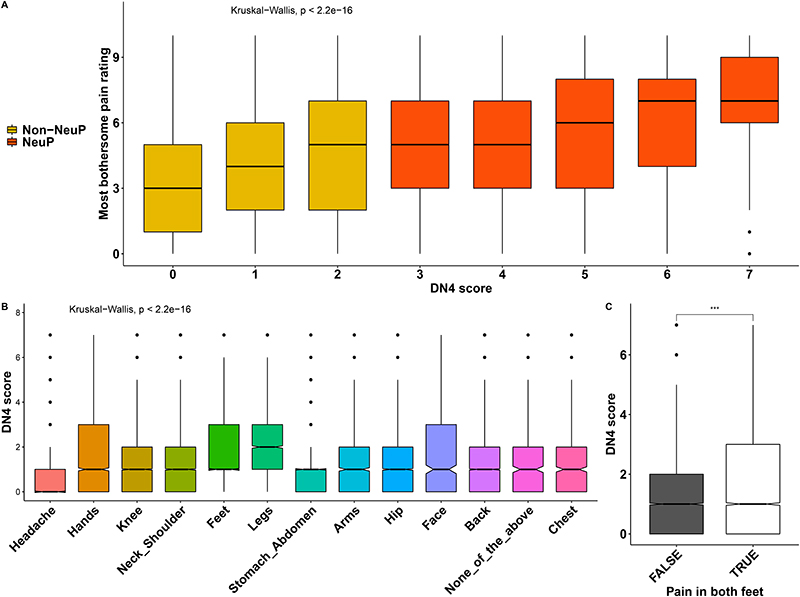
Pain rating and DN4 scores. A: Boxplots show the distribution of pain ratings for the most bothersome locations across DN4 scores for both painful groups. Group is colour coded. B: Boxplots show the DN4 score for all different locations of self-reported most bothersome pain. C: DN4 scores for people with and without pain in both feet, Omnibus Kruskall-Wallis tests are labelled in the plot. P.value < 0.001 is coded as ***, p.value < 0.01 is coded as ** 0.01, p.value < 0.05 is coded as *.

**Figure 7:**
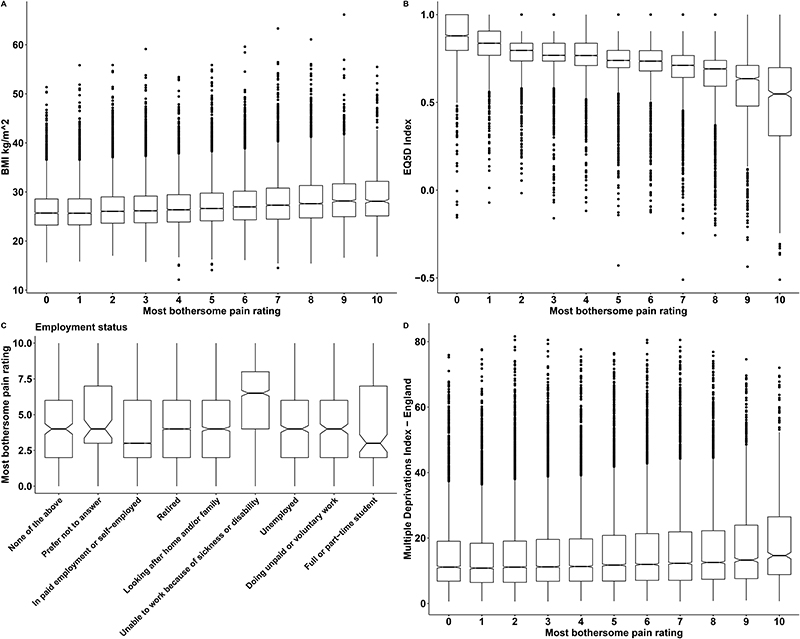
Intensity of pain versus participant characteristics. A: Boxplots show the distribution BMI for different self-reported pain intensities for the most bothersome location. B: Boxplots show the EQ5D index across self-reported pain intensities for the most bothersome locations. C: Frequencies of employment status across self-reported pain intensities for the most bothersome locations. D: Index of multiple deprivations or different self-reported pain intensities for the most bothersome location. Notched lines represent the median.

Focusing on people with chronic pain (both NeuP and Non-NeuP), we observed that DN4 scores were significantly associated with the pain severity rating and were higher for participants with higher pain ratings for the most bothersome pain, figure 6A. The highest DN4 scores were those of participants with the most bothersome pain in the feet and legs, followed by hands and face, figure 6B. People with pain in both feet had significantly higher DN4 scores than people with pain only in one foot, figure 6C. Pain severity ratings for the most bothersome location were higher in participants with higher BMI scores (figure 7A), lower quality of life (figure 7B), and/or who were unable to work due to sickness or disability (figure 7C) and slightly higher for among participants with higher deprivation scores (figure 7D). Pain in the feet and pain in the leg were moderately associated in participants with NeuP, supplementary figure 6.

### 3.3 Multivariable modelling

**Figure 8:**
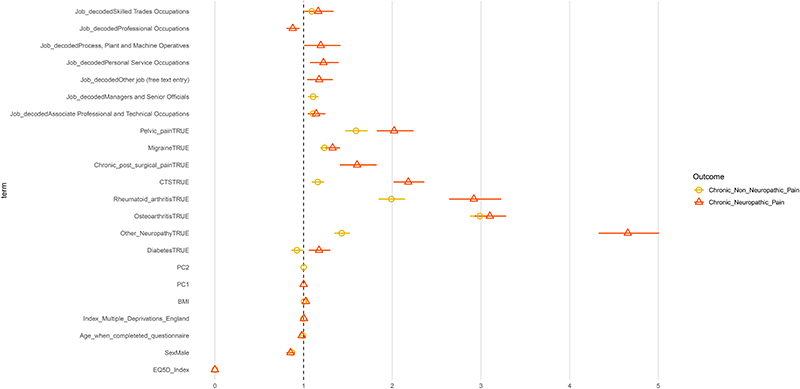
Coefficient estimates of the multinomial logit model. Dots represent the exponentiated coefficient estimates, i.e. odds ratios, and lines show the 95% confidence interval for all terms with an one-way ANOVA p.value < 0.05. Odds ratios are calculated for NeuP and Non-NeuP against the reference level NoCP.

In figure 8 we present the odds ratios (ORs) for the terms that reached significance for the multinomial model with a three-level dependent variable considering no chronic pain as the reference condition. All terms are in supplementary table 5. Both NeuP and non-neuP were strongly associated with having lower quality of life (assessed using EQ-5D index in which a higher score reflects a higher quality of life). The presence of diabetes increased the OR for NeuP and decreased them for Non-NeuP. All other self-reported conditions increased the ORs for both NeuP and Non-NeuP but had a larger effect for NeuP. Males had lower OR for both NeuP and Non-NeuP. In addition those who were “Managers and Senior Officials” were more likely to report Non-NeuP. “Process, Plant and Machine Operatives” and “Skilled Trades Occupations” increased the odds for NeuP, while “Professional Occupations” decreased the odds for NeuP.

**Figure 9:**
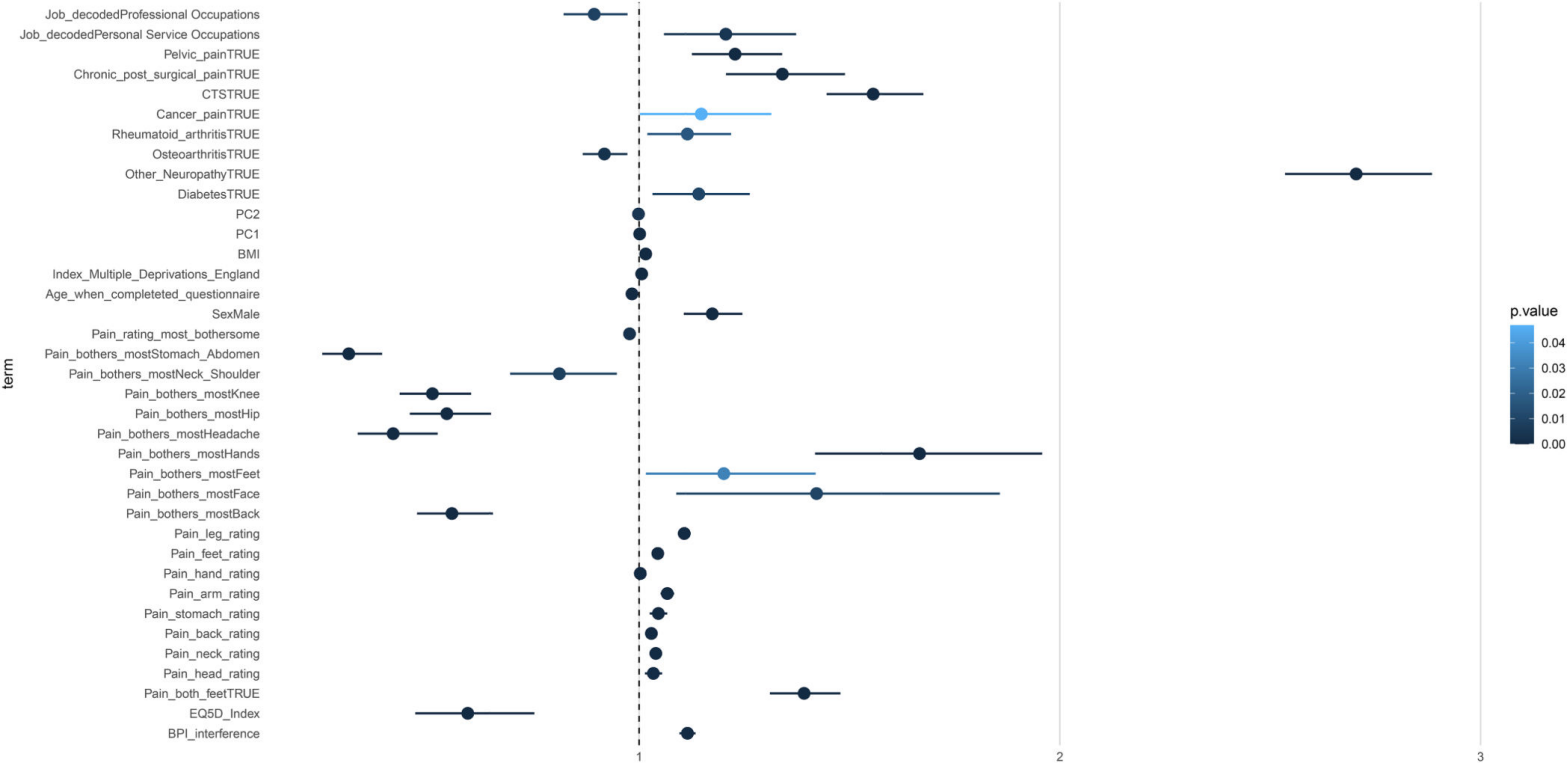
Coefficient estimates of the binomial logit model for the two painful groups. Dots represent the exponentiated coefficient estimates, i.e odds ratios, and lines show the 95% confidence interval for all terms with an one-way ANOVA p.value < 0.05. Odds ratios are calculated for NeuP against the reference level Non-NeuP. The p.value is colour coded.

In figure 9 and supplementary table 6 we present the ORs for a binomial model of NeuP versus Non-NeuP. Male participants had a higher OR for NeuP vs Non-NeuP. Higher BMI and lower age were associated with an increased OR for NeuP. Most bothersome pain in the feet was associated with increased odds for NeuP, whereas most bothersome pain in the abdomen, shoulder, knee, hip. Back, chest and headache was associated with reduced odds for NeuP. “Personal Service Occupations” was associated with increased odds for NeuP and “Professional Occupations” with decreased odds for NeuP.

## 4 Discussion

In this large cross-sectional study of middle-aged adults in UK Biobank, the prevalence of NeuP was 9.2%, making up 18.1% of those with chronic pain. The published range of prevalence estimates for NeuP in the general population is 3.2-17.9% [9,14,25,32,38,51,54,60–62]. In the UK, two studies reported similar prevalences of 8.2% [50] and 9.3% [48] respectively. The wide range of prevalences reported is partly due to differences in case ascertainment and definition. In Japan, the relatively low prevalence of 3.2% can be explained by the stringent case definition used [32]. In studies that used the same definition as the current one, the prevalence of NeuP was lower in France (6.9%; [9]) and higher in Canada (16.1% and 17.9%; [51,54]) and Morocco (10.6%; [25]), though these studies have a wider age range than the current study.

The main strength of this study is the NeuP sample size (n=13,747) which is more than 5 times that of the largest previous study in a community based cohort [14]. The usage of validated questionnaires allows direct comparison with other studies of NeuP, notably DOLORisk [27,40] and the Pain in Neuropathy Study (PiNS) [47].

The current study has enabled the identification of associations with a greater level of precision than previous studies. Additionally, unlike some previous studies we conducted multivariate regression analysis, which enabled us to control for covariates [14,25,32,48,51,61]. Although, in this study we have not done any causal inference or modelling and as such, results should only be interpreted as associations. First, people with NeuP have worse health-related quality of life than people without NeuP [3,32,49,51]. Most of the studies reporting this finding used only univariate hypothesis tests, which does not control for covariates, such as differing pain intensity [38,62], in contrast to the current study.

Second, this study supports previous findings that pain intensity is more severe in people with NeuP than people with non-NeuP [9,25,32,38,48,50,51,60,62]. Part of the possible reason for this is the relative difficulty in treating NeuP. It has previously been reported that significantly fewer people with NeuP rate their prescribed treatment as “very successful” [60].

Third, people with NeuP were more likely to report pain in their hands, legs or feet [9,25,32,50,62], whilst people with non-NeuP were more likely to report pain in their stomach or abdomen, neck or shoulder, knees, hips, head, chest and back [25,50,62]. Common aetiologies of NeuP including diabetes, surgery, carpal tunnel syndrome, complex regional pain syndrome and cancer often affect the upper and lower extremities. This is supported by the association of these comorbidities independently with NeuP.

In addition to these findings, we identified some novel associations with NeuP. This includes RA. Whilst arthritis is generally considered to be non-neuropathic in nature, there is growing evidence of a neuropathic component to this cause of pain [17,33,34,45]. Furthermore, RA can predispose to NeuP disorders such as carpal tunnel syndrome [58], vasculitic neuropathy [24] and spinal cord compression [15]. However, a previous study found no association with NeuP [22]. But, as individuals can suffer from pain in multiple locations, it is possible that rheumatoid arthritis could be present in a person with NeuP in another location with a different cause.

Another novel finding of this study is that BMI was significantly higher in people with NeuP than in people with non-NeuP. Despite not being previously reported in a community based cohort, this association has been reported in specific populations with NeuP including diabetic peripheral neuropathy [44] and rheumatoid arthritis [33]. Higher BMI is associated with increased risk of common conditions causing NeuP including diabetes [57] and cardiovascular disease [37], whilst obesity can place increased strain on the joints, exacerbating painful symptoms in rheumatoid arthritis [4].

Male sex was associated with increased risk of having NeuP versus non-NeuP, whereas female sex was associated with increased risk of having NeuP versus NoCP. Female sex has been consistently associated with NeuP when compared to non-NeuP in the literature, in both adjusted [9,50,60,62] and unadjusted analysis [25,48,51]. A notable exception is a Canadian study, which also used the DN4 [54].

Younger age was significantly associated with NeuP. Previous studies, mostly analysing age as a categorical variable, found that NeuP is more prevalent in older people [9,25,32,50,54], though some have reported no association [48,51,60]. A study conducted in France identified a peak prevalence of NeuP in the 50-64 year group [9]. As the age range of the cohort for the current study was 49-83 years (Q1 = 61, Q3 = 73) at the time of completing the chronic pain phenotyping survey, the age group expected to have the higher prevalence of NeuP were amongst the younger participants. This could explain these findings.

Being in a personal service, skilled trade or plant and machine operative job and not being in a professional occupation, were associated with NeuP. A previous study in France found that NeuP was significantly more prevalent in manual workers, farmers, retirees and other non-working people, compared to those in managerial positions [9]. These are occupations in which people are likely to require repetitive use of hands and feet, which could make nerve damage more likely.

This study also has some limitations. Our definition of NeuP relies on a self-completed screening tool for NeuP, which does not meet the grading system for “probable” or “definite” NeuP [19] as clinical examination is clearly not feasible in a population survey.

The study was cross-sectional, thus it was not possible to determine whether the relationships between NeuP and the independent variables reported are causal [31]. Moreover, demographic and socio-economic data were only collected during recruitment and are not concurrent with pain phenotyping. However, there are currently plans for UK Biobank to repeat the chronic pain survey in 2023 presenting an opportunity for future longitudinal studies.

Similarly, future studies will explore the wealth of genetic data within UK Biobank to validate recent findings and to potentially identify novel associations [55,56].

A further weakness of the study is that the UK Biobank had a very low initial response rate, is limited to white middle-to-oldaged adults and under-represents people from more socioeconomically deprived areas, as well as people who are obese, smoke, drink alcohol and self-report certain health conditions. It may also suffer from “healthy volunteer” bias [21]. However this should not make the exposure-disease associations not generaliseable [63]. Since NeuP has previously been associated with these health-related states, our estimate of the prevalence of NeuP may be conservative [10,33,39,44]. We also found an over-representation of females, younger age, lower BMI and less social deprivation in those participating in the pain phenotyping survey compared to those who did not participate, suggesting an element of ascertainment bias. However, it should be noted that whilst these observations reached significance statistically, this may reflect the fact that the study was highly powered to detect even small differences between groups.

In conclusion this study represents the largest epidemiological study of NeuP to date and confirms that the disorder is common and places a significantly greater burden on people compared to those without the disorder. In addition, it has identified demographics and clinical characteristics that increase the risk of having chronic NeuP, some of which may be amenable to targeted prevention. These findings will be of particular interest to healthcare professionals who will be able to influence clinical policy for preventing and treating the disorder.

## Supplementary Material

Supplementary Materials: figures, tables
