# Supplementary material for: Epidemiology of neuropathic pain: an analysis of prevalence and associated factors in UK Biobank
Source: Pain Rep. 2023 Feb 10;8(2):e1066. doi: 10.1097/PR9.0000000000001066 (PMC7614463; doi:10.1097/PR9.0000000000001066)
Supplement: SUPPLEMENTARY MATERIAL [file painreports-8-e1066-s001.pdf]

	Item No	Recommendation	Page No
**Title and abstract**	1	(*a*) Indicate the study's design with a commonly used term in the title or the abstract	1
		(*b*) Provide in the abstract an informative and balanced summary of what was done and what was found	2
**Introduction**			
Background/rationale	2	Explain the scientific background and rationale for the investigation being reported	3
Objectives	3	State specific objectives, including any prespecified hypotheses	3
**Methods**			
Study design	4	Present key elements of study design early in the paper	4
Setting	5	Describe the setting, locations, and relevant dates, including periods of recruitment, exposure, follow-up, and data collection	4
Participants	6	(*a*) Give the eligibility criteria, and the sources and methods of selection of participants	4
Variables	7	Clearly define all outcomes, exposures, predictors, potential confounders, and effect modifiers. Give diagnostic criteria, if applicable	4-5
Data sources/ measurement	8*	For each variable of interest, give sources of data and details of methods of assessment (measurement). Describe comparability of assessment methods if there is more than one group	4-5
Bias	9	Describe any efforts to address potential sources of bias	NA
Study size	10	Explain how the study size was arrived at	5
Quantitative variables	11	Explain how quantitative variables were handled in the analyses. If applicable, describe which groupings were chosen and why	5
Statistical methods	12	(*a*) Describe all statistical methods, including those used to control for confounding	5-6
		(*b*) Describe any methods used to examine subgroups and interactions	NA
		(*c*) Explain how missing data were addressed	6
		(*d*) If applicable, describe analytical methods taking account of sampling strategy	NA
		(*e*) Describe any sensitivity analyses	NA
**Results**			
Participants	13*	(a) Report numbers of individuals at each stage of study—eg numbers potentially eligible, examined for eligibility, confirmed eligible, included in the study, completing follow-up, and analysed	7
		(b) Give reasons for non-participation at each stage	NA
		(c) Consider use of a flow diagram	NA
Descriptive data	14*	(a) Give characteristics of study participants (eg demographic, clinical, social) and information on exposures and potential confounders	7
		(b) Indicate number of participants with missing data for each variable of interest	Table S2
Outcome data	15*	Report numbers of outcome events or summary measures	7

			Tables S3-S4
Main results	16	(*a*) Give unadjusted estimates and, if applicable, confounder-adjusted estimates and their precision (eg, 95% confidence interval). Make clear which confounders were adjusted for and why they were included	7-8<br>Figures 1-9<br>Table S5<br>Figure S3-S6
		(*b*) Report category boundaries when continuous variables were categorized	NA
		(*c*) If relevant, consider translating estimates of relative risk into absolute risk for a meaningful time period	NA
Other analyses	17	Report other analyses done—eg analyses of subgroups and interactions, and sensitivity analyses	NA
**Discussion**			
Key results	18	Summarise key results with reference to study objectives	9
Limitations	19	Discuss limitations of the study, taking into account sources of potential bias or imprecision. Discuss both direction and magnitude of any potential bias	11
Interpretation	20	Give a cautious overall interpretation of results considering objectives, limitations, multiplicity of analyses, results from similar studies, and other relevant evidence	9-10
Generalisability	21	Discuss the generalisability (external validity) of the study results	9-11
**Other information**			
Funding	22	Give the source of funding and the role of the funders for the present study and, if applicable, for the original study on which the present article is based	12

*Give information separately for exposed and unexposed groups.

Supplementary table 1: STROBE Statement—Checklist of items that should be included in reports of ***cross-sectional studies***

Note: An Explanation and Elaboration article discusses each checklist item and gives methodological background and published examples of transparent reporting. The STROBE checklist is best used in conjunction with this article (freely available on the Web sites of PLoS Medicine at http://www.plosmedicine.org/, Annals of Internal Medicine at http://www.annals.org/, and Epidemiology at http://www.epidem.com/). Information on the STROBE Initiative is available at www.strobe-statement.org.

Variable	Missing values percentages %
BPI_severity	0.7
BPI_interference	0.8
EQ5D_Index	0
EQ5D_Health_today	0.2
Pain_both_feet	65
Pain_head_rating	0.2
Pain_face_rating	0.2
Pain_neck_rating	0.2
Pain_back_rating	0.2
Pain_stomach_rating	0.3
Pain_hip_rating	0.4
Pain_knee_rating	0.2
Pain_arm_rating	0.2
Pain_hand_rating	0.2
Pain_feet_rating	0.2
Pain_leg_rating	0.3
Pain_chest_rating	0.3
Pain_bothers_most	17.4
Pain_rating_most_bothersome	18.2
Sex	0
Ethnic_background	0
Age_when_completeted_questionnaire	0
Index_Multiple_Deprivations_England	12.9
Index_Multiple_Deprivations_Wales	96.2
Index_Multiple_Deprivations_Scotland	93.5
BMI	0.3
Employment_status	0
PC1	2
PC2	2
PC3	2
Diabetes	0.3
Other_Neuropathy	2.6
Fibromyalgia	2.6
CFS_ME	0
Osteoarthritis	3.9
Rheumatoid_arthritis	5.2
Cancer_pain	0.7
CTS	2.4

CRPS	6.4
Chronic_post_surgical_pain	1
Gout	0.4
Migraine	0.5
Pelvic_pain	1.1
Job_decoded	27.4
ICD_decoded_toplevel	61.3
Ethnic_background_grouped	1

Supplementary table 2: Percentages of missing values for all variables considered in both the descriptive hypothesis testing and multivariable modelling.

Outcome level	Coefficient	Odds-Ratio	std.error	statistic	df	p.value	95%CI.low	95%CI.high
Non-NeuP	(Intercept)	2814.8467	0.1776	44.7135	941.5233	<0.0001	1986.3568	3988.8916
Non-NeuP	EQ5D_Index	1.00E-04	0.0725	-130.7751	105520.5909	<0.0001	1.00E-04	1.00E-04
Non-NeuP	SexMale	0.8741	0.0171	-7.8598	103496.7438	<0.0001	0.8453	0.9039
Non-NeuP	Age_when_completeted_questionnaire	0.9932	0.0011	-6.1637	100056.8677	<0.0001	0.9911	0.9954
Non-NeuP	Index_Multiple_Deprivations_England	0.9969	7.00E-04	-4.2404	1176.757	<0.0001	0.9955	0.9984
Non-NeuP	BMI	1.0044	0.0019	2.2899	98869.0434	0.022	1.0006	1.0081
Non-NeuP	DiabetesYes	0.9238	0.0367	-2.1611	69964.2693	0.0307	0.8598	0.9927
Non-NeuP	Other_NeuropathyYes	1.4164	0.0312	11.1409	54693.5849	<0.0001	1.3322	1.5058
Non-NeuP	OsteoarthritisYes	2.9804	0.0196	55.5868	14365.2578	<0.0001	2.8678	3.0974
Non-NeuP	Rheumatoid_arthritisYes	1.9707	0.0393	17.2457	9026.8154	<0.0001	1.8244	2.1286
Non-NeuP	CTSYes	1.154	0.0311	4.6071	25941.8873	<0.0001	1.0858	1.2264
Non-NeuP	CRPSYes	2.2147	0.0982	8.0986	7613.2815	<0.0001	1.827	2.6848
Non-NeuP	MigraineYes	1.2372	0.0203	10.4721	74672.0766	<0.0001	1.1889	1.2875
Non-NeuP	Pelvic_painYes	1.5624	0.0408	10.9325	50868.6053	<0.0001	1.4422	1.6925
Non-NeuP	Job_decodedAssociate Professional and Technical Occupations	1.1006	0.0274	3.5057	107514.9586	5.00E-04	1.0432	1.1613
Non-NeuP	Job_decodedManagers and Senior Officials	1.1056	0.0281	3.5731	106232.821	4.00E-04	1.0464	1.1682
NeuP	(Intercept)	3693.1546	0.2664	30.8299	2994.4386	<0.0001	2190.352	6227.0312
NeuP	EQ5D_Index	<0.0001	0.1029	-114.0615	100771.4724	<0.0001	0	0
NeuP	SexMale	0.858	0.0291	-5.2579	98512.6293	<0.0001	0.8105	0.9084
NeuP	Age_when_completeted_questionnaire	0.9788	0.0019	-11.5564	93625.0376	<0.0001	0.9753	0.9824
NeuP	Index_Multiple_Deprivations_England	1.0043	0.0011	3.8351	1300.2144	1.00E-04	1.0021	1.0065
NeuP	BMI	1.0266	0.0029	9.2005	87282.312	0	1.0209	1.0324
NeuP	PC3	0.9974	0.0013	-2.0472	1672.1068	0.0408	0.9949	0.9999
NeuP	DiabetesYes	1.1591	0.0533	2.7669	62976.3419	0.0057	1.044	1.2868
NeuP	Other_NeuropathyYes	4.5463	0.0374	40.4863	34719.3456	<0.0001	4.2249	4.8921
NeuP	FibromyalgiaYes	1.9595	0.1388	4.8478	6243.2144	<0.0001	1.4928	2.572
NeuP	OsteoarthritisYes	3.0978	0.0295	38.3093	12973.2608	<0.0001	2.9237	3.2823
NeuP	Rheumatoid_arthritisYes	2.8414	0.0511	20.4439	8466.822	<0.0001	2.5707	3.1407
NeuP	CTSYes	2.1374	0.0411	18.4717	8976.3611	<0.0001	1.9719	2.3169
NeuP	CRPSYes	3.1239	0.1109	10.2717	3008.3319	<0.0001	2.5134	3.8827
NeuP	Chronic_post_surgical_painYes	1.5081	0.0672	6.1154	25026.9388	<0.0001	1.3221	1.7204
NeuP	MigraineYes	1.3124	0.0318	8.5515	75644.3979	<0.0001	1.2331	1.3968
NeuP	Pelvic_painYes	1.9406	0.0525	12.6392	50596.0959	<0.0001	1.751	2.1507
NeuP	Job_decodedAssociate Professional and Technical Occupations	1.1302	0.0448	2.7299	103102.6599	0.0063	1.0351	1.234
NeuP	Job_decodedOther job (free text entry)	1.1685	0.0634	2.4582	105447.1891	0.014	1.0321	1.323
NeuP	Job_decodedPersonal Service Occupations	1.2148	0.0679	2.8656	102021.6271	0.0042	1.0634	1.3878
NeuP	Job_decodedProcess, Plant and Machine Operatives	1.1903	0.0878	1.9838	98729.7843	0.0473	1.0021	1.4138
NeuP	Job_decodedProfessional Occupations	0.8823	0.0427	-2.9306	98581.298	0.0034	0.8115	0.9594
NeuP	Job_decodedSkilled Trades Occupations	1.1515	0.0706	1.9974	102830.383	0.0458	1.0026	1.3224

Supplementary Table 3: Multinomial regression table, showing the Odds-ratio (i.e. exponentiated coefficient estimate), standard error of the estimate, F-statistic, p.value, lower and upper point of the 95% confidence interval for each coefficient and level of the outcome compared to NoCP.

Coefficient	Odds-ratio	std.error	statistic	df	p.value	95%CI. low	95%CI. high
(Intercept)	0.5102	0.2873	-2.3416	1670.3511	0.0193	0.2904	0.8965
BPI_severity	1.0531	0.0094	5.515	25616.6125	<0.0001	1.0339	1.0726
EQ5D_Index	0.3757	0.1115	-8.7822	40523.5925	<0.0001	0.3019	0.4674
Pain_head_rating	1.0321	0.0105	3.0194	35994.279	0.0025	1.0112	1.0535
Pain_neck_rating	1.0319	0.0072	4.3822	43648.5639	<0.0001	1.0175	1.0465
Pain_back_rating	1.0245	0.0068	3.5842	34253.0164	3e-04	1.011	1.0382
Pain_stomach_rating	1.0426	0.0102	4.0752	43433.2413	<0.0001	1.0219	1.0637
Pain_arm_rating	1.0629	0.0078	7.7764	39565.9359	<0.0001	1.0467	1.0793
Pain_hand_rating	1.0543	0.0068	7.8247	38910.8099	<0.0001	1.0404	1.0684
Pain_feet_rating	1.0408	0.0066	6.0281	38292.5995	<0.0001	1.0274	1.0544
Pain_leg_rating	1.1099	0.007	14.9353	41074.079	<0.0001	1.0948	1.1252
Pain_chest_rating	1.0482	0.0154	3.0536	39214.9791	0.0023	1.017	1.0803
Pain_bothers_mostBack	0.5877	0.0829	-6.41	44144.5543	<0.0001	0.4995	0.6914
Pain_bothers_mostChest	0.7397	0.1467	-2.056	43434.0549	0.0398	0.5548	0.986
Pain_bothers_mostFeet	1.2937	0.0858	3.0017	43991.5507	0.0027	1.0935	1.5305
Pain_bothers_mostHands	1.6423	0.0845	5.8715	44439.3329	<0.0001	1.3917	1.9381
Pain_bothers_mostHeadache	0.4467	0.1164	-6.9237	44203.7701	<0.0001	0.3556	0.5612
Pain_bothers_mostHip	0.574	0.0905	-6.1334	43223.3115	<0.0001	0.4807	0.6854
Pain_bothers_mostKnee	0.5494	0.0852	-7.0269	44396.6958	<0.0001	0.4648	0.6493
Pain_bothers_mostLegs	1.2354	0.09	2.3488	43983.1425	0.0188	1.0356	1.4738
Pain_bothers_mostNeck_Shoulder	0.8431	0.08	-2.1335	44208.5026	0.0329	0.7208	0.9862
Pain_bothers_mostStomach_Abdomen	0.3374	0.1168	-9.299	43461.1107	<0.0001	0.2683	0.4242
SexMale	1.1405	0.0302	4.353	43941.609	<0.0001	1.075	1.2101
Age_when_completeted_questionnaire	0.9822	0.0021	-8.5646	40239.005	<0.0001	0.9782	0.9863
Index_Multiple_Deprivations_England	1.0058	0.0011	5.203	2195.2878	<0.0001	1.0036	1.0079
BMI	1.0185	0.0028	6.4725	39298.6866	<0.0001	1.0129	1.0242
DiabetesYes	1.1439	0.0518	2.5962	37805.9533	0.0094	1.0335	1.2661
Other_NeuropathyYes	2.7268	0.0334	29.9946	3948.4265	<0.0001	2.5537	2.9116
FibromyalgiaYes	1.3459	0.1076	2.7598	4060.3957	0.0058	1.0898	1.6621
Rheumatoid_arthritisYes	1.1445	0.0447	3.0193	3253.6347	0.0026	1.0484	1.2493
Cancer_painYes	1.1649	0.0697	2.1889	18764.395	0.0286	1.0161	1.3355
CTSYes	1.5821	0.0383	11.9724	3884.971	<0.0001	1.4676	1.7055
CRPSYes	1.2119	0.075	2.5644	662.7275	0.0106	1.0461	1.4041
Chronic_post_surgical_painYes	1.2969	0.0546	4.7569	16342.2931	<0.0001	1.1651	1.4435
Pelvic_painYes	1.2249	0.0447	4.5417	33827.7733	<0.0001	1.1222	1.337
Job_decodedPersonal Service Occupations	1.1983	0.0668	2.7082	44034.8416	0.0068	1.0512	1.3659
Job_decodedProfessional Occupations	0.9115	0.0435	-2.1312	43641.6093	0.0331	0.837	0.9926

Supplementary Table 3: Binomial regression table, showing the Odds-ratio (i.e. exponentiated coefficient estimate), standard error of the estimate, F-statistic, p.value, lower and upper point of the 95% confidence interval for each coefficient and level of the outcome compared to NoNeuP.

Outcome level	Coefficient	Odds-Ratio	std.error	statistic	df	p.value	95%CI.low	95%CI.high
Non-NeuP	(Intercept)	3168.7056	0.1106	72.8825	93098.7709	0	2551.1416	3935.7656
Non-NeuP	EQ5D_Index	1e-04	0.072	-132.1335	108450.665	0	1e-04	1e-04
Non-NeuP	SexMale	0.8709	0.017	-8.14	108454.6323	0	0.8423	0.9003
Non-NeuP	Age_when_completeted_questionnaire	0.9936	0.0011	-5.8428	103985.3144	0	0.9915	0.9958
Non-NeuP	Index_Multiple_Deprivations_England	0.9967	7e-04	-4.5383	616.9022	0	0.9952	0.9981
Non-NeuP	BMI	1.0045	0.0019	2.3587	97124.9724	0.0183	1.0008	1.0082
Non-NeuP	PC1	0.9999	2e-04	-0.6013	2075.5792	0.5477	0.9995	1.0003
Non-NeuP	PC2	1.0009	4e-04	2.3322	7742.7331	0.0197	1.0001	1.0016
Non-NeuP	PC3	0.9987	7e-04	-1.9062	24281.9178	0.0566	0.9974	1
Non-NeuP	DiabetesYes	0.925	0.0362	-2.1565	91118.9498	0.031	0.8617	0.9929
Non-NeuP	Other_NeuropathyYes	1.4308	0.0312	11.4916	28005.0457	0	1.346	1.5209
Non-NeuP	OsteoarthritisYes	2.9879	0.0194	56.3186	26129.6535	0	2.8762	3.1039
Non-NeuP	Rheumatoid_arthritisYes	1.9897	0.0384	17.92	38324.3792	0	1.8455	2.1452
Non-NeuP	Cancer_painYes	0.9134	0.0563	-1.6078	83772.2984	0.1079	0.8179	1.02
Non-NeuP	CTSYes	1.16	0.0309	4.8034	29858.8799	0	1.0918	1.2324
Non-NeuP	Chronic_post_surgical_painYes	1.0899	0.0549	1.567	70401.8988	0.1171	0.9786	1.2137
Non-NeuP	GoutYes	0.9504	0.0334	-1.5237	81579.7685	0.1276	0.8902	1.0147
Non-NeuP	MigraineYes	1.2353	0.0202	10.4767	97881.1912	0	1.1874	1.2851
Non-NeuP	Pelvic_painYes	1.5906	0.0405	11.4677	71283.8068	0	1.4693	1.7219
Non-NeuP	Job_decodedAssociate Professional and Technical Occupations	1.1064	0.0272	3.7165	107085.4579	2e-04	1.049	1.1671
Non-NeuP	Job_decodedElementary Occupations	0.9671	0.0583	-0.5729	108107.3232	0.5667	0.8626	1.0843
Non-NeuP	Job_decodedManagers and Senior Officials	1.1072	0.028	3.6405	106786.3284	3e-04	1.0481	1.1696
Non-NeuP	Job_decodedOther job (free text entry)	1.0414	0.0394	1.0289	108941.3233	0.3035	0.964	1.1249
Non-NeuP	Job_decodedPersonal Service Occupations	1.0045	0.0439	0.1024	108494.1135	0.9184	0.9217	1.0947
Non-NeuP	Job_decodedProcess, Plant and Machine Operatives	1.0091	0.0562	0.1612	105490.8968	0.8719	0.9039	1.1265
Non-NeuP	Job_decodedProfessional Occupations	1.0263	0.0251	1.0358	107232.7643	0.3003	0.9771	1.0781
Non-NeuP	Job_decodedSales and Customer Service Occupations	0.9614	0.058	-0.6786	107311.6693	0.4974	0.858	1.0772
Non-NeuP	Job_decodedSkilled Trades Occupations	1.0913	0.0436	2.0053	108078.0842	0.0449	1.002	1.1886
NeuP	(Intercept)	3241.9169	0.1708	47.3415	78473.5954	0	2319.8007	4530.5726
NeuP	EQ5D_Index	0	0.1018	-116.1352	103015.0281	0	0	0
NeuP	SexMale	0.8533	0.0288	-5.5013	103717.8456	0	0.8064	0.9029
NeuP	Age_when_completeted_questionnaire	0.9797	0.0018	-11.1976	102336.4745	0	0.9762	0.9832
NeuP	Index_Multiple_Deprivations_England	1.0041	0.0011	3.5969	684.2529	3e-04	1.0019	1.0063
NeuP	BMI	1.0262	0.0028	9.1207	80840.6591	0	1.0205	1.0319
NeuP	PC1	1.0008	3e-04	2.4405	3048.1131	0.0147	1.0002	1.0014
NeuP	PC2	0.9991	6e-04	-1.5641	9594.8839	0.1178	0.998	1.0002
NeuP	PC3	0.9984	0.0011	-1.5339	3892.2445	0.1251	0.9963	1.0004
NeuP	DiabetesYes	1.1743	0.0527	3.0498	48597.6737	0.0023	1.0591	1.302
NeuP	Other_NeuropathyYes	4.6561	0.0373	41.2119	14174.0174	0	4.3277	5.0096
NeuP	OsteoarthritisYes	3.1007	0.0293	38.6382	13212.7843	0	2.9277	3.2839
NeuP	Rheumatoid_arthritisYes	2.9201	0.0512	20.9298	3184.5565	0	2.6412	3.2285
NeuP	Cancer_painYes	1.0697	0.0751	0.8977	47893.4286	0.3693	0.9233	1.2393
NeuP	CTSYes	2.1808	0.0409	19.0776	7253.6466	0	2.0129	2.3627
NeuP	Chronic_post_surgical_painYes	1.6035	0.066	7.1589	39987.194	0	1.4091	1.8248

NeuP	GoutYes	1.0669	0.0506	1.28	75824.4907	0.2005	0.9662	1.1781
NeuP	MigraineYes	1.3274	0.0315	8.9876	67810.9491	0	1.2479	1.412
NeuP	Pelvic_painYes	2.0213	0.052	13.5263	34248.5389	0	1.8253	2.2383
NeuP	Job_decodedAssociate Professional and Technical Occupations	1.1421	0.0444	2.9906	107085.112	0.0028	1.0468	1.246
NeuP	Job_decodedElementary Occupations	1.1536	0.0884	1.6167	106705.2078	0.1059	0.9701	1.3717
NeuP	Job_decodedManagers and Senior Officials	1.0623	0.0465	1.2986	98616.3421	0.1941	0.9697	1.1637
NeuP	Job_decodedOther job (free text entry)	1.1752	0.063	2.5628	102390.5566	0.0104	1.0387	1.3297
NeuP	Job_decodedPersonal Service Occupations	1.2236	0.0671	3.0088	105495.8796	0.0026	1.0729	1.3955
NeuP	Job_decodedProcess, Plant and Machine Operatives	1.1937	0.0872	2.0306	105729.8969	0.0423	1.0062	1.4162
NeuP	Job_decodedProfessional Occupations	0.8777	0.0424	-3.0771	104467.1828	0.0021	0.8077	0.9537
NeuP	Job_decodedSales and Customer Service Occupations	1.1152	0.0902	1.2091	105291.7376	0.2266	0.9345	1.3307
NeuP	Job_decodedSkilled Trades Occupations	1.1654	0.07	2.188	106760.555	0.0287	1.0161	1.3367

Supplementary Table 5: Multinomial regression table, showing the Odds-ratio (i.e. exponentiated coefficient estimate), standard error of the estimate, F-statistic, p.value, lower and upper point of the 95% confidence interval for each coefficient and level of the outcome compared to NoCP.

Coefficient	Odds-ratio	std.error	statistic	df	p.value	95%CI. low	95%CI. high
(Intercept)	0.0219	89.3293	-0.0428	44428.9411	0.9659	0	2.400279984015 62e+74
BPI_interference	1.1147	0.0087	12.4998	28520.7077	0	1.0959	1.1339
EQ5D_Index	0.5926	0.1207	-4.3345	36474.9412	0	0.4677	0.7508
Pain_both_feetYes	1.392	0.0306	10.8028	276.722	0	1.3106	1.4784
Pain_head_rating	1.0338	0.0101	3.2905	36962.4267	0.001	1.0135	1.0544
Pain_neck_rating	1.0396	0.0072	5.3602	24723.3153	0	1.0249	1.0544
Pain_back_rating	1.029	0.0067	4.2402	41284.9231	0	1.0155	1.0427
Pain_stomach_rating	1.0457	0.0101	4.4125	37318.3834	0	1.0251	1.0666
Pain_hip_rating	1.0061	0.0073	0.8328	26164.3183	0.405	0.9918	1.0207
Pain_knee_rating	1.0025	0.0068	0.3604	33485.0296	0.7185	0.9892	1.0159
Pain_arm_rating	1.0669	0.0078	8.3463	30801.1842	0	1.0508	1.0833
Pain_hand_rating	1.0027	3e-04	9.4325	39304.9013	0	1.0022	1.0033
Pain_feet_rating	1.0442	0.0066	6.5169	29388.8668	0	1.0307	1.0579
Pain_leg_rating	1.1071	0.007	14.5356	40425.3694	0	1.092	1.1224
Pain_bothers_mostBack	0.5549	0.0825	-7.1394	40648.5034	0	0.4721	0.6523
Pain_bothers_mostChest	0.7963	0.1363	-1.6712	41786.4873	0.0947	0.6097	1.0401
Pain_bothers_mostFace	1.4217	0.1364	2.5798	41953.8425	0.0099	1.0882	1.8573
Pain_bothers_mostFeet	1.201	0.0853	2.1469	39942.8204	0.0318	1.0161	1.4196
Pain_bothers_mostHands	1.6664	0.0823	6.2047	38645.8338	0	1.4181	1.9581
Pain_bothers_mostHeadache	0.4151	0.1159	-7.5867	41871.4635	0	0.3307	0.521
Pain_bothers_mostHip	0.5428	0.0901	-6.7788	37456.0134	0	0.4549	0.6477
Pain_bothers_mostKnee	0.5085	0.0849	-7.9634	37315.3271	0	0.4305	0.6006
Pain_bothers_mostLegs	1.1644	0.0898	1.6954	35795.2838	0.09	0.9765	1.3884
Pain_bothers_mostNeck_Shoulder	0.8102	0.0796	-2.6434	37663.2418	0.0082	0.6931	0.947
Pain_bothers_mostNone_of_the_above	0.9298	0.1586	-0.4586	40479.4144	0.6465	0.6814	1.2689
Pain_bothers_mostStomach_Abdomen	0.3096	0.1164	-10.0697	40274.2505	0	0.2464	0.389
Pain_rating_most_bothersome	0.9772	0.0078	-2.9632	16130.3913	0.003	0.9623	0.9922
SexMale	1.1736	0.0302	5.3033	38996.9144	0	1.1062	1.2452
Age_when_completeted_questionnaire	0.9829	0.0021	-8.2476	40632.4797	0	0.9789	0.987
Index_Multiple_Deprivations_England	1.0059	0.0011	5.2174	814.0039	0	1.0037	1.0082
BMI	1.0156	0.0028	5.45	27565.7455	0	1.0099	1.0212
Employment_statusIn paid employment or self-employed	8332.3661	162.8997	0.0554	45149.407	0.9558	0	3.845727951257 53e+142
Employment_statusRetired	14.4952	89.3292	0.0299	44427.8163	0.9761	0	1.586502294819 4e+77

Employment_statusLooking after home and/or family	0.0017	156.556	-0.0408	44902.8759	0.9674	0	3.07982503636743e+130
Employment_statusUnable to work because of sickness or disability	0.0012	173.2959	-0.0387	45087.2178	0.9691	0	3.98188685008653e+144
Employment_statusUnemployed	0.0021	169.96	-0.0362	44979.0112	0.9711	0	1.00775685832708e+142
PC1	1.0012	3e-04	3.5701	1637.2867	4e-04	1.0005	1.0018
PC2	0.9984	6e-04	-2.8523	7386.7698	0.0044	0.9972	0.9995
PC3	0.999	0.0011	-0.8742	2830.2793	0.3821	0.9969	1.0012
DiabetesYes	1.1414	0.0515	2.5678	27460.3238	0.0102	1.0318	1.2627
Other_NeuropathyYes	2.7042	0.0329	30.2096	6831.1254	0	2.5352	2.8845
OsteoarthritisYes	0.9173	0.0295	-2.9226	5448.9415	0.0035	0.8657	0.972
Rheumatoid_arthritisYes	1.1145	0.0454	2.3877	1265.1049	0.0171	1.0195	1.2183
Cancer_painYes	1.1474	0.0692	1.9876	24567.5123	0.0469	1.0019	1.3141
CTSYes	1.5562	0.0376	11.7554	9477.7188	0	1.4456	1.6753
Chronic_post_surgical_painYes	1.3403	0.0538	5.4477	27234.8381	0	1.2062	1.4893
GoutYes	1.0706	0.0494	1.3808	40745.5462	0.1673	0.9718	1.1796
MigraineYes	1.0461	0.0316	1.4273	34348.7934	0.1535	0.9833	1.113
Pelvic_painYes	1.2278	0.0446	4.6065	23655.9685	0	1.1252	1.3399
Job_decodedAssociate Professional and Technical Occupations	1.0724	0.0448	1.5601	42718.0167	0.1187	0.9822	1.1709
Job_decodedElementary Occupations	1.0931	0.0882	1.0089	37783.1826	0.313	0.9195	1.2993
Job_decodedManagers and Senior Officials	0.9645	0.0475	-0.7615	43222.7221	0.4464	0.8788	1.0585
Job_decodedOther job (free text entry)	1.1299	0.0633	1.9292	42899.9057	0.0537	0.9981	1.2792
Job_decodedPersonal Service Occupations	1.2058	0.0662	2.8254	44781.0815	0.0047	1.059	1.373
Job_decodedProcess, Plant and Machine Operatives	1.1292	0.0885	1.3731	43487.555	0.1697	0.9494	1.3429
Job_decodedProfessional Occupations	0.893	0.0434	-2.609	43040.4405	0.0091	0.8202	0.9722
Job_decodedSales and Customer Service Occupations	1.0926	0.0897	0.9873	44179.4399	0.3235	0.9164	1.3027
Job_decodedSkilled Trades Occupations	1.013	0.071	0.1824	44516.8543	0.8553	0.8814	1.1643
Employment_statusDoing unpaid or voluntary work	17612.3272	220.744	0.0443	33508.7184	0.9647	0	1.41321867208904e+192

Supplementary Table 6: Binomial regression table, showing the Odds-ratio (i.e. exponentiated coefficient estimate), standard error of the estimate, F-statistic, p.value, lower and upper point of the 95% confidence interval for each coefficient and level of the outcome compared to NoNeuP.

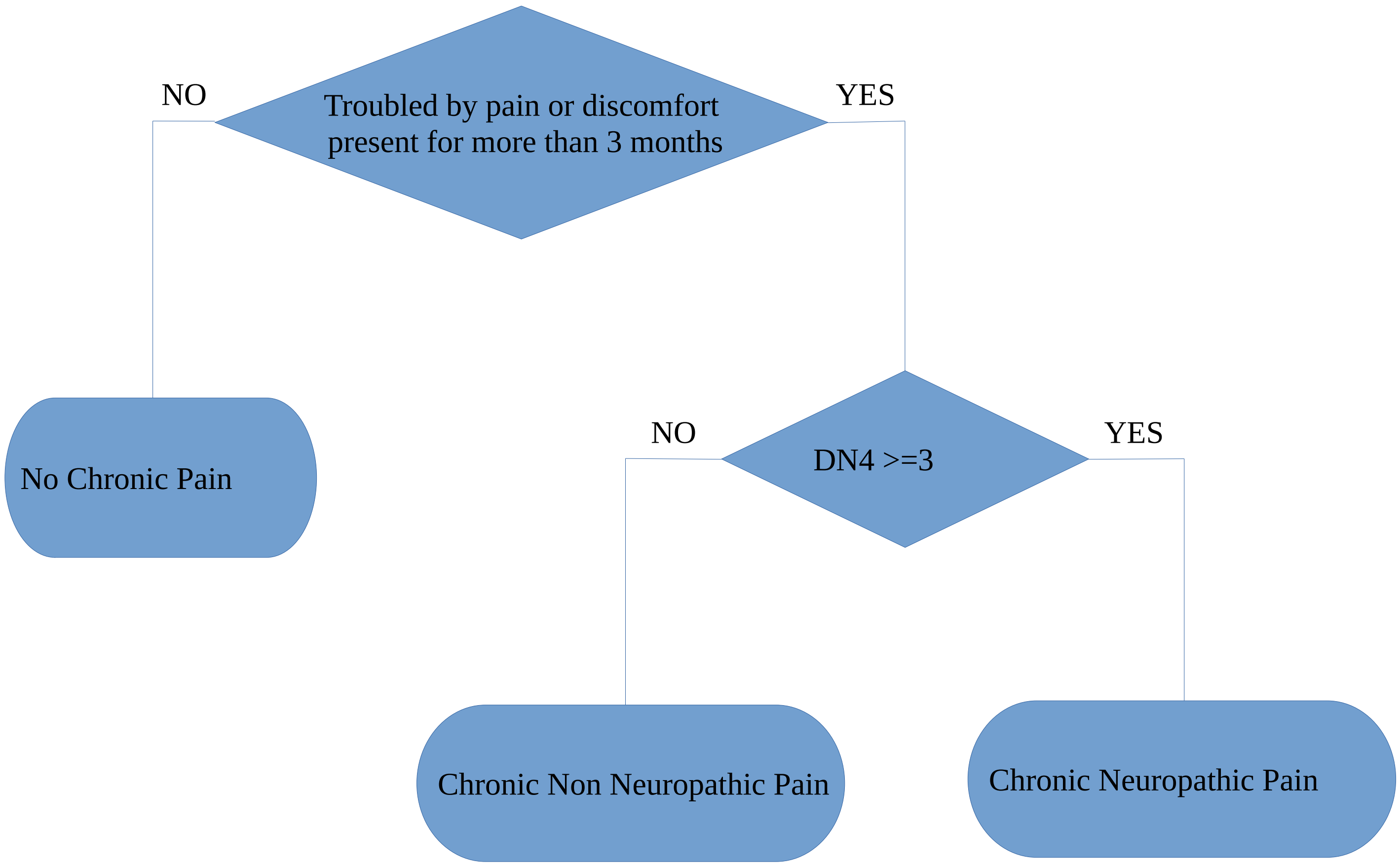

Supplementary Figure 1: Flowchart of the group definitions

A

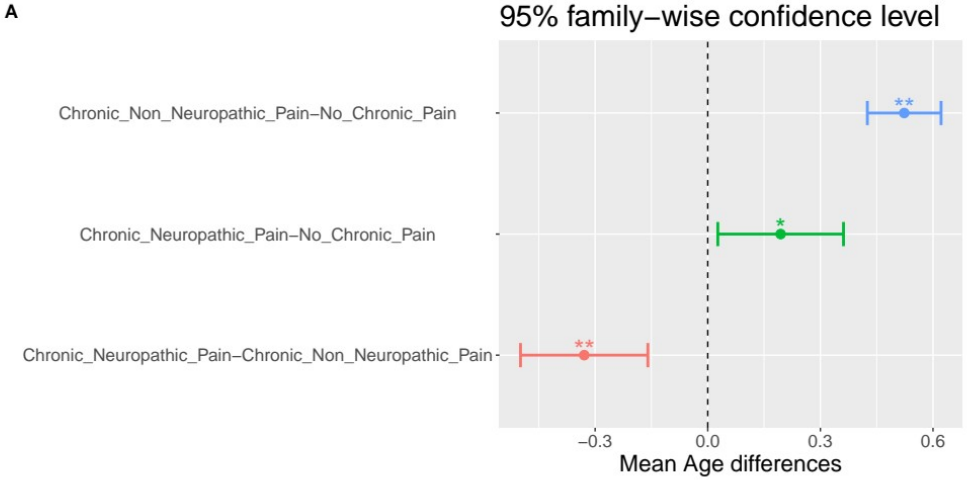

B

	Dimension	Value	No_Chronic_Pain	Chronic_Non_Neuropathic_Pain	Chronic_Neuropathic_Pain
1	Female	Residuals	−26.0536308503014	21.0077953868443	9.19748932152672
2	Female	p values	<0.0001	<0.0001	<0.0001
3	Male	Residuals	26.0536308503014	−21.0077953868443	−9.19748932152672
4	Male	p values	<0.0001	<0.0001	<0.0001

C

	Dimension	Value	No_Chronic_Pain	Chronic_Non_Neuropathic_Pain	Chronic_Neuropathic_Pain
1	BAME	Residuals	5.19248137209141	−6.25787700321982	1.702877
2	BAME	p values	<0.0001	<0.0001	0.797300
3	Mixed	Residuals	−0.273187108541649	−1.11060879532308	2.369724
4	Mixed	p values	1	1	0.160200
5	White	Residuals	−4.34149950107226	5.97008443773198	−2.683978
6	White	p values	1e−04	<0.0001	0.065500

D

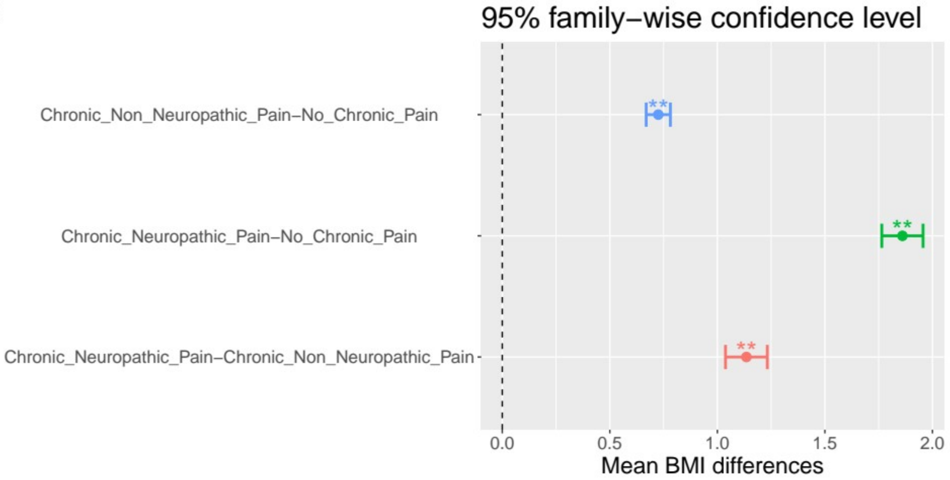

Supplementary Figure 2: Post-hoc follow-up tests for omnibus tests shown in figure 1. A: Tukey's HSD mean differences in age for all outcome levels. B-C: Chi-square post-hoc test for all levels of outcome. Standardised residuals and Bonferroni corrected p.values. D: Tukey's HSD mean differences in BMI for all outcome levels. P.value < 0.01 is coded as **, p.value < 0.05 is coded as *.

A

	Dimension	Value	No_Chronic_Pain	Chronic_Non_Neuropathic_Pain	Chronic_Neuropathic_Pain
1	None of the above	Residuals	−0.327081121073469	0.250561684392678	0.137935074413102
2	None of the above	p values	1	1	1
3	Prefer not to answer	Residuals	−0.206330431246903	−0.165256411545335	0.638350660308744
4	Prefer not to answer	p values	1	1	1
5	In paid employment or self−employed	Residuals	11.6826503432546	−8.57611719491137	−5.56352762780595
6	In paid employment or self−employed	p values	<0.0001	<0.0001	<0.0001
7	Retired	Residuals	−8.00963341878631	8.29953370940934	−0.311923644905685
8	Retired	p values	<0.0001	<0.0001	1
9	Looking after home and/or family	Residuals	−0.250071215886216	0.49184451569524	−0.406589959050507
10	Looking after home and/or family	p values	1	1	1
11	Unable to work because of sickness or disability	Residuals	−18.1700576096873	2.16591721574574	27.7050269714519
12	Unable to work because of sickness or disability	p values	<0.0001	0.8186	<0.0001
13	Unemployed	Residuals	−0.593032244873146	−0.633739826311685	2.10546923052035
14	Unemployed	p values	1	1	0.9518
15	Doing unpaid or voluntary work	Residuals	−0.0248588747275635	0.752431245317658	−1.2401319209818
16	Doing unpaid or voluntary work	p values	1	1	1
17	Full or part−time student	Residuals	−0.587864992638074	0.935158206195278	−0.578834825035822
18	Full or part−time student	p values	1	1	1

B

	Dimension	Value	No_Chronic_Pain	Chronic_Non_Neuropathic_Pain	Chronic_Neuropathic_Pain
1	Administrative and Secretarial Occupations	Residuals	−3.45542067652593	2.30006977	2.08487811030977
2	Administrative and Secretarial Occupations	p values	0.0165	0.64330000	1
3	Associate Professional and Technical Occupations	Residuals	−5.53490219946127	3.68965473	3.33025434240247
4	Associate Professional and Technical Occupations	p values	<0.0001	0.00670000	0.026
5	Elementary Occupations	Residuals	−2.21409957396008	−0.77697194	5.21835473971792
6	Elementary Occupations	p values	0.8047	1.00000000	<0.0001
7	Managers and Senior Officials	Residuals	3.00323198538079	−1.51558778	−2.64603045083972
8	Managers and Senior Officials	p values	0.0801	1.00000000	0.2443
9	Other job (free text entry)	Residuals	−1.9061375132423	−0.31329329	3.87912224946455
10	Other job (free text entry)	p values	1	1.00000000	0.0031
11	Personal Service Occupations	Residuals	−5.54841949824402	1.26530281	7.53579966711134
12	Personal Service Occupations	p values	<0.0001	1.00000000	<0.0001
13	Process, Plant and Machine Operatives	Residuals	0.0658633365120532	−2.38637197	4.00099234342252
14	Process, Plant and Machine Operatives	p values	1	0.51050000	0.0019
15	Professional Occupations	Residuals	10.1660420174022	−2.90477059	−12.7958417781712
16	Professional Occupations	p values	<0.0001	0.11030000	<0.0001
17	Sales and Customer Service Occupations	Residuals	−1.90989895159743	−0.03016651	3.39733305480865
18	Sales and Customer Service Occupations	p values	1	1.00000000	0.0204
19	Skilled Trades Occupations	Residuals	−1.76524280298689	−0.12115468	3.30090893774506
20	Skilled Trades Occupations	p values	1	1.00000000	0.0289

C

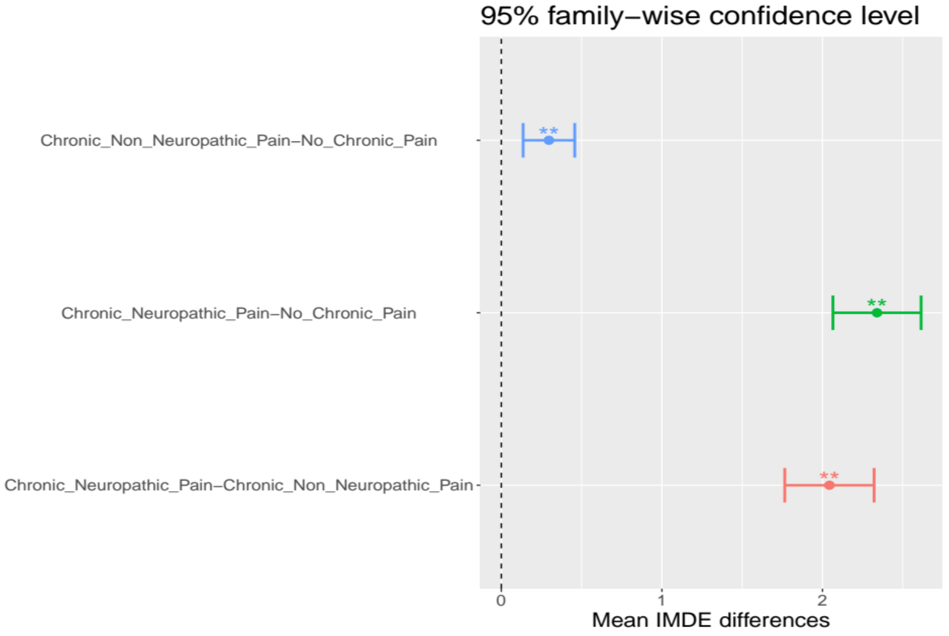

Supplementary Figure 3: Post-hoc follow-up tests for omnibus tests shown in figure 2. A-B: Chi-square post-hoc test for all levels of outcome. Standardised residuals and Bonferroni corrected p.values. C: Tukey's HSD mean differences in Index of Multiple Deprivations England for all outcome levels. P.value < 0.01 is coded as **, p.value < 0.05 is coded as *.

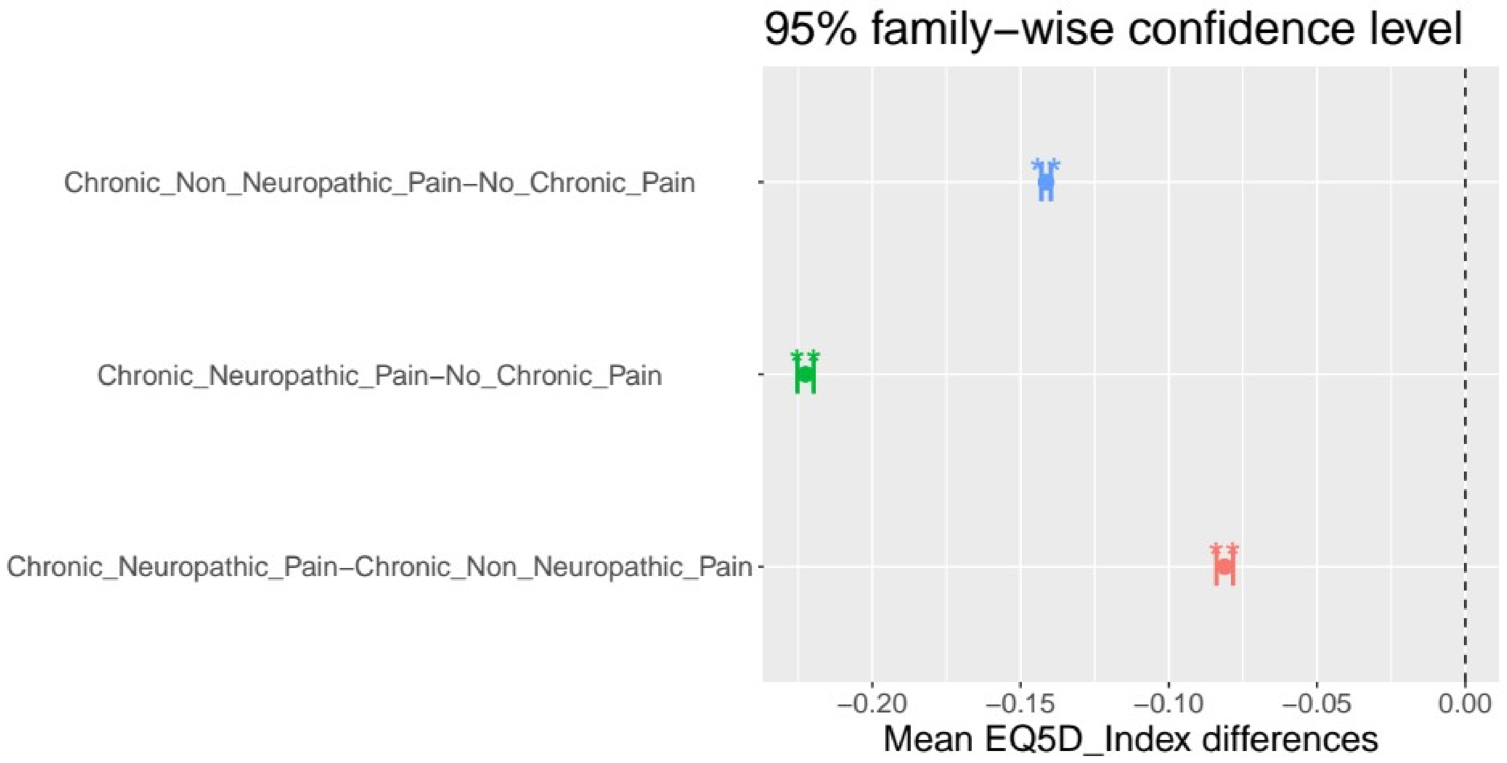

Supplementary Figure 4: Post-hoc follow-up tests for the omnibus test shown in figure 4. Tukey’s HSD mean differences in EQ5D index for all outcome levels. P.value < 0.01 is coded as **, p.value < 0.05 is coded as *.

	Dimension	Value	No_Chronic_Pain	Chronic_Non_Neuropathic_Pain	Chronic_Neuropathic_Pain
1	Certain infectious and parasitic diseases	Residuals	−6.24558356437805	1.63265848097847	7.63709009572292
2	Certain infectious and parasitic diseases	p values	<0.0001	1	<0.0001
3	Congenital malformations, deformations and chromosomal abnormalities	Residuals	0.859157712708666	0.212161082802247	−1.76552109462882
4	Congenital malformations, deformations and chromosomal abnormalities	p values	1	1	1
5	Diseases of the blood and blood−forming organs and certain disorders involving the immune mechanism	Residuals	−0.00150148242788975	−1.74144137606679	2.85313336123517
6	Diseases of the blood and blood−forming organs and certain disorders involving the immune mechanism	p values	1	1	0.221
7	Diseases of the circulatory system	Residuals	−2.29288019876535	2.43308043491678	−0.197944754718154
8	Diseases of the circulatory system	p values	1	0.764	1
9	Diseases of the digestive system	Residuals	−0.0790725858358351	0.631691680636868	−0.903522331311575
10	Diseases of the digestive system	p values	1	1	1
11	Diseases of the ear and mastoid process	Residuals	−0.48612048950299	−0.274590505072392	1.25193723195031
12	Diseases of the ear and mastoid process	p values	1	1	1
13	Diseases of the genitourinary system	Residuals	2.47968065279676	−0.259588789615659	−3.66830950437928
14	Diseases of the genitourinary system	p values	0.671	1	0.012
15	Diseases of the musculoskeletal system and connective tissue	Residuals	−1.5406468838129	2.47716551610639	−1.51183200135762
16	Diseases of the musculoskeletal system and connective tissue	p values	1	0.675	1
17	Diseases of the nervous system	Residuals	−17.2849205307284	6.70678166699038	17.5537859958716
18	Diseases of the nervous system	p values	<0.0001	<0.0001	<0.0001
19	Diseases of the respiratory system	Residuals	−0.00254857535184033	−0.299341708461126	0.494214788381761
20	Diseases of the respiratory system	p values	1	1	1
21	Diseases of the skin and subcutaneous tissue	Residuals	2.45385220196519	−2.13561380395345	−0.554712403461019
22	Diseases of the skin and subcutaneous tissue	p values	0.721	1	1
23	Endocrine, nutritional and metabolic diseases	Residuals	−3.35896475927119	2.18268478031015	1.97174153248822
24	Endocrine, nutritional and metabolic diseases	p values	0.04	1	1
25	Factors influencing health status and contact with health services	Residuals	0.888106889690421	−0.163318862440593	−1.19866549815287
26	Factors influencing health status and contact with health services	p values	1	1	1
27	Injury, poisoning and certain other consequences of external causes	Residuals	8.49707399928666	−4.79740244772017	−6.17312505625288
28	Injury, poisoning and certain other consequences of external causes	p values	<0.0001	<0.0001	<0.0001
29	Neoplasms	Residuals	2.46182688832086	−1.31915597149751	−1.90437809624796
30	Neoplasms	p values	0.705	1	1
31	Pregnancy, childbirth and the puerperium	Residuals	9.23875141293393	−4.92373849934394	−7.19061642162847
32	Pregnancy, childbirth and the puerperium	p values	<0.0001	<0.0001	<0.0001
33	Symptoms, signs and abnormal clinical and laboratory findings, not elsewhere classified	Residuals	5.69978623394203	−2.65669600669285	−5.0598351373456
34	Symptoms, signs and abnormal clinical and laboratory findings, not elsewhere classified	p values	<0.0001	0.402	<0.0001

Supplementary Figure 5: Chi-square post-hoc test for all levels of outcome. Standardised residuals and Bonferroni corrected p.values.

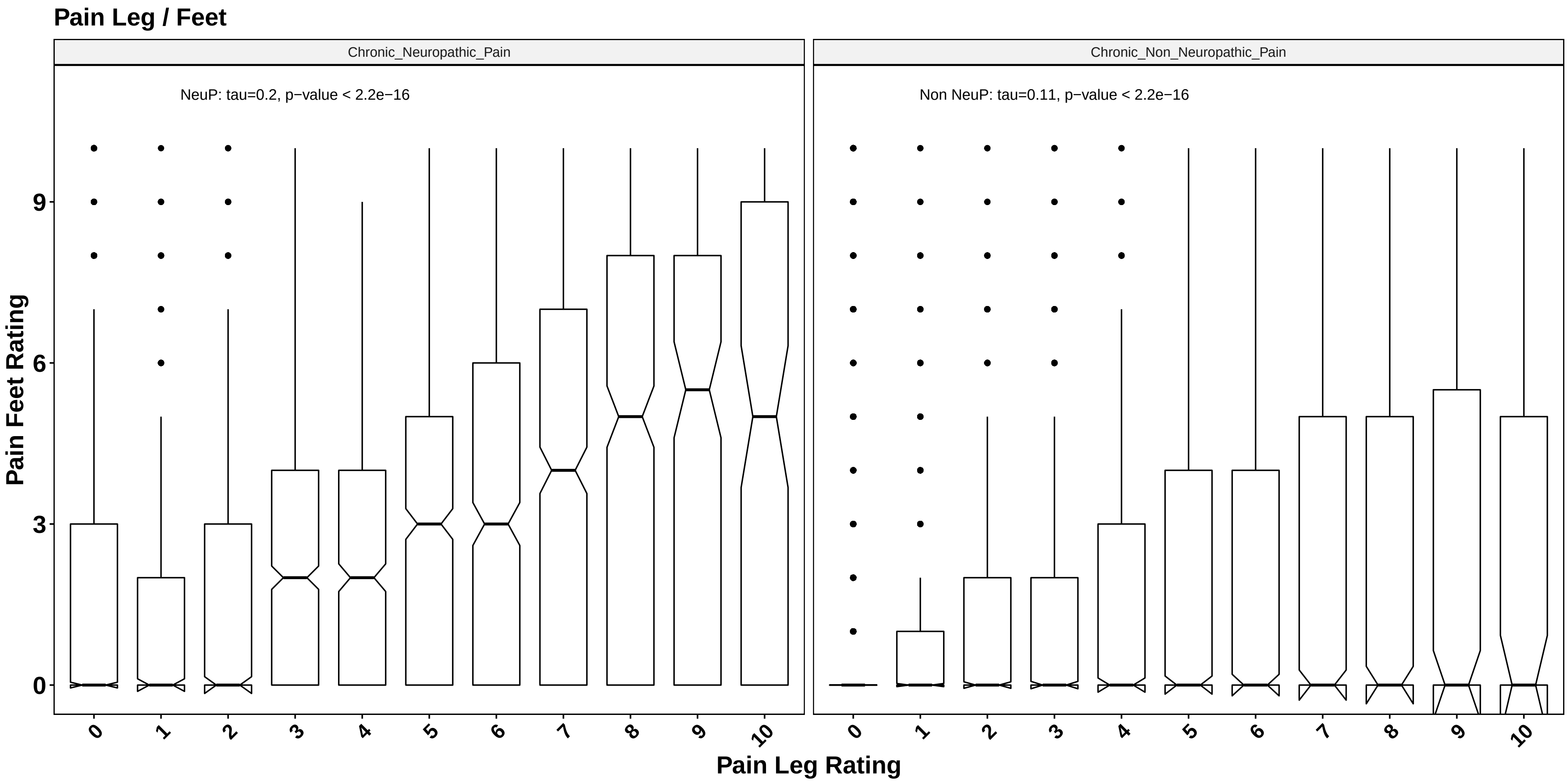

Supplementary Figure 6: Pain in the feet and leg associations for participants with NeuP and Non-NeuP. Associations were quantified using Kendal's Tau correlation.
